# TRIM32 regulates insulin sensitivity by controlling insulin receptor degradation in the liver

**DOI:** 10.1038/s44319-024-00348-7

**Published:** 2025-01-02

**Authors:** Shilpa Thakur, Priya Rawat, Budheswar Dehury, Prosenjit Mondal

**Affiliations:** 1 School of Biosciences and Bioengineering, Indian Institute of Technology Mandi, Mandi, 175005 H.P. India; 2 Department of Bioinformatics, Manipal School of Life Sciences, Manipal Academy of Higher Education, Manipal, 576104 India; 3 Department of Biological Sciences, Indian Institute of Science Education and Research Berhampur (IISER Berhampur), Berhampur, Odisha 760010 India

**Keywords:** Insulin Receptor, Insulin Resistance, Ubiquitination, SREBP-1c, TRIM32, Metabolism, Post-translational Modifications & Proteolysis

## Abstract

Impaired insulin receptor signaling is strongly linked to obesity-related metabolic conditions like non-alcoholic fatty liver disease (NAFLD) and Type 2 diabetes (T2DM). However, the exact mechanisms behind impaired insulin receptor (INSR) signaling in obesity induced by a high-fat diet remain elusive. In this study, we identify an E3 ubiquitin ligase, tripartite motif-containing protein 32 (TRIM32), as a key regulator of hepatic insulin signaling that targets the insulin receptor (INSR) for ubiquitination and proteasomal degradation in high-fat diet (HFD) mice. HFD induces the nuclear translocation of SREBP-1c (Sterol Regulatory Element-Binding Protein 1c), resulting in increased expression of TRIM32 in hepatocytes. TRIM32 ubiquitylates INSR and facilitates its proteasomal degradation, leading to severe insulin resistance and fat accumulation within the liver of high-fat diet induced obese (DIO) mice. Conversely, liver-specific knockdown of TRIM32 enhances INSR expression and hepatic insulin sensitivity. Reduced AMPK signaling and phosphorylation of SREBP-1c at S372 in high-fat DIO mice promotes the nuclear translocation of SREBP-1c, leading to increased TRIM32 expression. In conclusion, our results demonstrate that TRIM32 promotes diet-induced hepatic insulin resistance by targeting the INSR to degradation.

## Synopsis

E3 ubiquitin ligase TRIM32 targets the insulin receptor for ubiquitin-mediated degradation, resulting in high-fat diet-induced hepatic insulin resistance.

- High-fat diet induces SREBP-1c-dependent expression of the E3 ubiquitin ligase TRIM32.
- TRIM32 impairs cellular insulin action by degrading cell surface insulin receptors in the liver.
- Hepatic downregulation of TRIM32 increases INSR expression and increases hepatic and systemic insulin sensitivity in high-fat diet-fed mice.

## Abstract (web summary)

E3 ubiquitin ligase TRIM32 targets the insulin receptor for ubiquitin-mediated degradation, resulting in high-fat diet-induced hepatic insulin resistance.

## Graphical abstract

**Figure:**
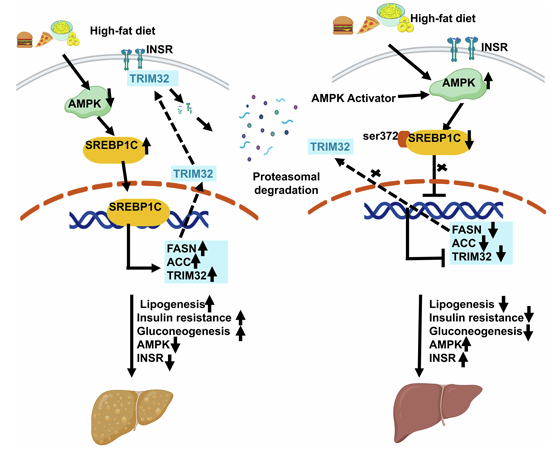

**Figure:**
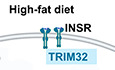

## Introduction

The insulin receptor (INSR) is the critical player in the insulin signaling axis. Impaired insulin receptor function and its downregulation are the fundamental cause of several metabolic disorders, including T2DM, NAFLD, Obesity, etc (Liu et al, 2022; Li et al, 2022). The INSR interacts with insulin at the cell surface and mediates the downstream signaling. Mutations in the INSR gene result in metabolic disorders, and the decrease in both INSR and its post-receptor components is strongly associated with insulin resistance (Okabayashi et al, 1989; Krook and O’Rahilly, 1996). NAFLD, diabetes, and obesity show reductions in the overall amount of insulin receptors and the specific fraction found on cell membranes (Sesti et al, 2001). Restoring INSR levels through genetic or drug-based approaches holds promise as a potential treatment avenue (Wang et al, 2019). Simultaneously, efforts have been directed toward uncovering endogenous regulators of INSR that influence its behavior and signaling under both physiological and pathophysiological conditions. Several major mechanisms, including mitochondrial dysfunction, oxidative stress, inflammation, INSR mutations, endoplasmic reticulum stress, and E3 ubiquitin ligases, can regulate cell surface INSR expression and signaling (Ronald Kahn, 1978). Therefore, targeting any of these conditions provides a therapeutic approach to the treatment of obesity and obesity-driven metabolic disease conditions.

Several E3 ubiquitin ligases are well known as negative regulators of insulin signaling (Yaribeygi et al, 2019; Yang et al, 2016). The canonical model for INSR ubiquitination involves insulin-dependent recruitment of the E3 ligase, facilitating endocytosis and endosomal sorting to attenuate signaling from the activated INSR (Nagarajan et al, 2016). Recent research has highlighted the potential role of ubiquitin-mediated protein degradation in developing insulin resistance and its associated disorders. The tripartite motif-containing (TRIM) protein family, a group of E3 ubiquitin ligases, has emerged as a crucial player in this process (Zhang et al, 2023). Studies have shown that specific TRIM proteins are key regulators in metabolic disorders, including type 2 diabetes, obesity, non-alcoholic fatty liver disease, and atherosclerosis (Xu et al, 2008; Yang et al, 2016). Their influence extends to multiple signaling pathways and cellular processes, such as insulin and peroxisome proliferator-activated receptor signaling, glucose and lipid metabolism, inflammatory responses, and cell cycle regulation.

Here, we provide comprehensive evidence linking TRIM family member TRIM32 to insulin resistance and related metabolic disorders. Here, our results define a new pathway regulating INSR biological activity and demonstrate the potential utility of modulating this pathway for therapeutic benefit. We performed an RNA sequencing analysis and identified Tripartite motif protein 32 (TRIM32) as a potent and previously unidentified inhibitor of insulin receptor expression. However, the mechanism of its action is entirely undefined for insulin receptor dynamics. Our study revealed that hepatic TRIM32 is regulated by insulin via SREBP-1c in the HFD-fed mouse model. Using both in vitro and in vivo approaches, we provide evidence that TRIM32 plays a critical role in ubiquitinating INSR, targeting it for proteasomal degradation.

## Results

### The degradation of the hepatic insulin receptors primarily mediates high-fat diet-induced insulin resistance

**Figure 1:**
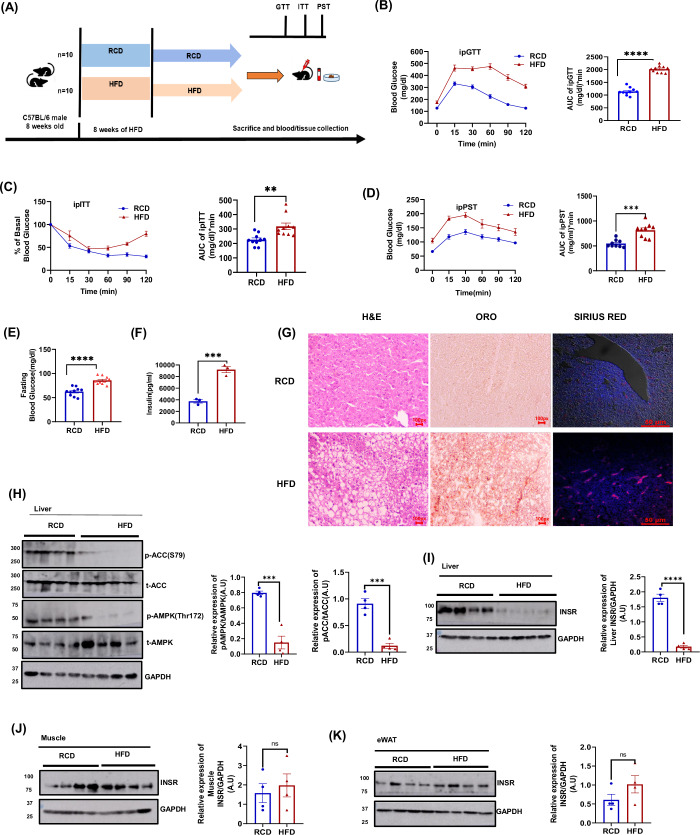
High-fat diet-induced insulin resistance is primarily mediated by the degradation of hepatic insulin receptors. (**A**) Representation of dietary regimen plan of RCD and HFD group (*n* = 10). (**B**) Graphical representation of ipGTT (*n* = 10 mice each). Mice were fasted for 6 h after 2 h of the dark cycle, and 2 g/kg body weight Glucose was injected intraperitoneally. Glucose levels were measured before Glucose administration and at 15, 30, 60, 90, and 120 min post-injection (left). AUC for the ipGTT curve (right). (**C**) Graphical representation of ipITT data (*n* = 10 mice each). Mice were fasted for 6 h after 2 h of the dark cycle, and 0.5 I.U/kg of body weight of insulin was injected intraperitoneally. Glucose levels were measured before and at 15, 30, 60, 90, and 120 min post-injection. AUC for the ipITT curve. (**D**) Graphical representation of ipPST (*n* = 10 mice each). Mice fasted for 12 h before 2 h of the dark cycle, and 100 mg/kg of body weight pyruvate was administrated intraperitoneally. Glucose levels were measured before Glucose administration and at 15, 30, 60, 90, and 120 min post-injection. AUC for the ipPST curve. (**E**) Graphical representation of Fasting blood glucose measured after overnight fasting (*n* = 10). (**F**) Fasting systemic Insulin levels measured after overnight fasting (*n* = 3), biological replicates. (**G**) Representation of H&E staining, Oil red O, and Sirius Red of RCD and HFD mice liver. All images were taken at 40x. Scale bar (50 µm). (**H**) Qualitative and quantitative representation of AMPKα and ACC phosphorylation at Thr172 and S79 residue of mice liver (*n* = 4), biological replicates. (**I**) Qualitative and quantitative representation of INSR levels from whole liver lysate of RCD and HFD mice (*n* = 4), biological replicates. (**J**) Qualitative and quantitative representation of INSR levels from Muscle of RCD, HFD, group (*n* = 4), biological replicates. (**K**) Qualitative and quantitative representation of INSR levels from eWAT of RCD, HFD, group (*n* = 4), biological replicates. Graphs were plotted in Graph pad prism8. The statistical significance was assessed by unpaired two-tailed t-test, and data has been expressed as Mean ± SEM. *p < 0.05, **p < 0.01, ***p < 0.001 and ****p < 0.0001. Data information: In (**B**), unpaired two-tailed t-test, *p* = 0.0001 (AUC of ipGTT of RCD vs. HFD), (**C**) AUC of ipITT of RCD vs. HFD (*p* = 0.0021), (**D**) AUC of ipPST of RCD vs. HFD (*p* = 0.0001), (**E**) fasting blood RCD vs. HFD (*p* = 0.0001), (**F**) Fasting insulin RCD vs. HFD (*p* = 0.0009), (**H**) RCD vs. HFD (*p* = 0.0003), pACC RCD vs. HFD (*p* = 0.0003), (**I**) RCD vs. HFD (*p* = 0.0001), (**J**) RCD vs. HFD (*p* = 0.6206), (**K**) RCD vs. HFD (*p* = 0.1801). Source data are available online for this figure.

We developed a high-fat-diet-fed obese mice model (Fig. 1A) to provide a deeper understanding to unravel the endogenous mechanisms underlying obesity-induced insulin resistance. Mice were maintained on a regular chow and high-fat diet for 8 weeks. We performed metabolic tests, including ipGTT, ipITT, and ipPST (Fig. 1B–D, respectively), to monitor the impact of HFD on glucose excursions and insulin sensitivity. Our findings indicated that a high-fat diet leads to basal hyperglycemia and hyperinsulinemia (Fig. 1E,F). Consistent with earlier research, our findings showed that HFD led to impaired glucose tolerance and reduced insulin sensitivity (Guo, 2014) (Fig. 1B,C, respectively). Further, we found that HFD significantly augments hepatic glucose production, as observed in the pyruvate stimulation test (Fig. 1D). It shows that HFD-mediated reduced insulin sensitivity affects the regulation of hepatic gluconeogenesis in the HFD group. We have analyzed the liver-to-body weight ratio (Fig. EV1A), which shows increased liver weight due to a high-fat diet. Consistently, HFD-fed mice exhibited dramatically higher serum cholesterol, TG, AST, and ALT levels with higher liver Cholesterol and TG as compared with RCD mice (Fig. EV1B–G, respectively). These data indicate that HFD is sufficient to aggravate dyslipidemia and hepatotoxicity. Histological analysis of the HFD liver H&E sections revealed hepatic inflammatory response, hepatic ballooning, microvesicular, and macrovesicular steatosis (Fig. 1G). Oil red O staining further reveals increased hepatic lipid accumulation (Fig. 1G). Sirius red staining shows hepatic fibrosis (Fig. 1G). Consistent with earlier research, our findings reinforce that HFD led to dyslipidemia and hepatotoxicity. Dysregulated hepatic de novo lipogenesis (DNL) has been identified as one of the determinant processes in the development of NAFLD.

**Figure EV1:**

High-fat diet-induced insulin resistance is primarily mediated by the degradation of hepatic insulin receptors. (**A**) Graphical representation of normalized Liver to body weight ratio (*n* = 10). (**B**–**E**) Graphical representation of serum cholesterol, triglyceride, AST, and ALT levels, respectively (*n* = 3), biological replicates. (**F**, **G**) Graphical representation of Liver Triglyceride and Cholesterol data, respectively (*n* = 3), biological replicates. (**H**–**K**) Graphical representation of hepatic ballooning, macrovesicular & microvesicular steatosis, and NAFLD score (*n* = 3), biological replicates. Graphs were plotted in Graph pad prism8. The statistical significance was assessed by unpaired two-tailed t-test, and data has been expressed as Mean ± SEM. *p < 0.05, **p < 0.01, ***p < 0.001, and ****p < 0.0001. Data information: In (1**A**) unpaired two-tailed t-test, *p* = 0.0252 (RCD vs. HFD), (**B**) RCD vs. HFD (*p* = 0.034), (**C**) RCD vs. HFD (*p* = 0.0414), (**D**) AST ratio of RCD vs. HFD (*p* = 0.0420), (**E**) ALT ratio of RCD vs. HFD (*p* = 0.0009), (**F**) Liver Cholesterol RCD vs. HFD (*p* = 0.0019), (**G**) Liver TG of RCD vs. HFD (*p* = 0.0125), (**H**) RCD vs. HFD (*p* < 0.0001), (**I**) RCD vs HFD (*p* < 0.0001), (**J**) RCD vs HFD (*p* < 0.0001), (**K**) RCD vs HFD (*p* < 0.0001). Source data are available online for this figure.

AMPK activation and inactivation of acetyl CoA carboxylase (ACC) can slow the DNL process. To investigate the potential effect of HFD on AMPK activity, we visualized the phosphorylation of Thr172 residue of AMPK (p-AMPK), a marker of AMPK activity; our data suggested that HFD diminished AMPK activity in the liver, as evidenced by p-AMPK(Thr172) and its downstream target acetyl-CoA carboxylase (p-ACC, Ser79/221) (Fig. 1H). These findings indicate that HFD can augment the DNL process by reducing AMPK signaling in HFD mice.

It has been known that HFD induces insulin resistance by modulating several insulin signaling pathway proteins (Kido et al, 2000; Yi et al, 2013; Rui et al, 2002). To determine the effect of HFD on insulin sensitivity in classical energy storage tissues such as the liver, muscle, and adipose tissue, we checked the expression of the insulin signaling protein, Insulin receptor(INSR). Western blotting data revealed that HFD led to a significant downregulation of INSR expression, specifically in the liver of HFD-fed mice (Fig. 1I), not in skeletal muscle (Fig. 1J) or epididymal White Adipose Tissue (eWAT) (Fig. 1K). Based on our data, we assumed that decreased INSR protein levels in the liver were associated with the development of insulin resistance. This indicates that HFD-induced systemic insulin resistance and liver-specific INSR downregulation were sufficient to developing impaired glycemia and fatty liver phenotype in HFD-fed DIO mice. We conclude that HFD-induced insulin resistance primarily involves the degradation of hepatic insulin receptors. Next, we tried to find out the mechanism of how HFD downregulates INSR expression, specifically in the liver.

### HFD induces TRIM32 expression to promote hepatic insulin receptor degradation

**Figure 2:**
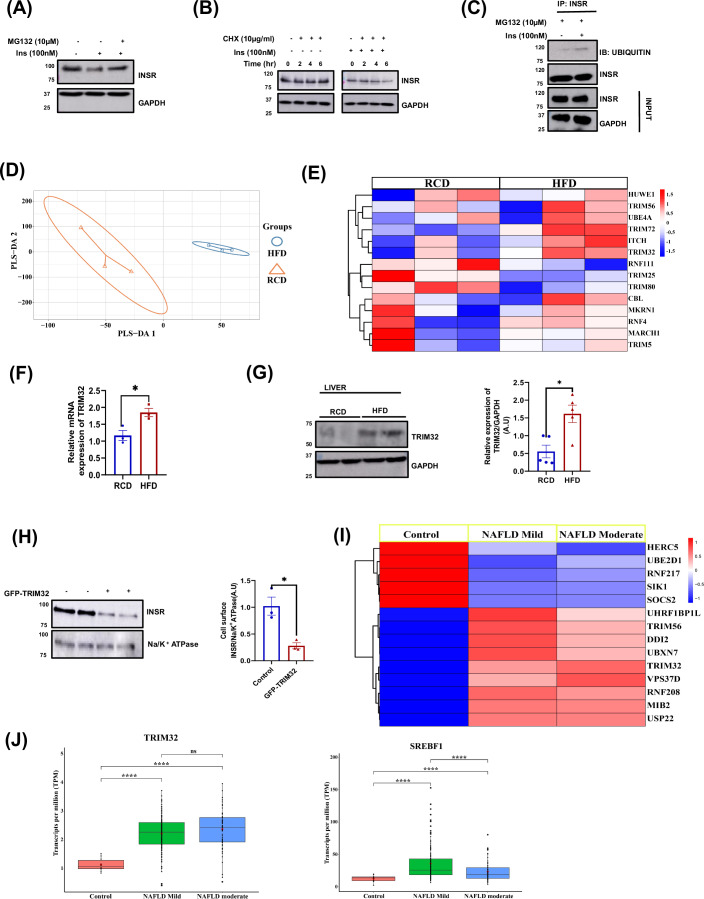
HFD induces TRIM32 expression to promote hepatic insulin receptor ubiquitination and degradation. (**A**) Western blot showing insulin receptor degradation in the presence of insulin (100 nM) and proteasomal inhibitor MG-132 (10 µM) treatment for 24 h. (**B**) Insulin receptor degradation was assessed after cells were treated with 100 nM insulin for 24 h. CHX (100 µg /ml) treatment was given after 18 h of insulin treatment, i.e., for 6 h, 4 h, 2 h, and 0 h before the completion of 24 h, and INSR expression was analyzed using WB. (**C**) INSR was pulled down after 24 h of insulin (100 nM) and MG132 (10 µM) treatment in mouse primary hepatocytes and analyzed for ubiquitination using WB. (**D**) PLS-DA plot of RNA-seq from mice’s liver shows both groups are well segregated, and the expression of a differentially expressed set of genes from the HFD and RCD groups of mice. (**E**) Clustered Heat map showing differentially expressed ubiquitin ligases of RCD and HFD mice liver (*n* = 3), biological replicates. (**F**) Graphical representation of RT-PCR data of TRIM32 gene from the liver of RCD and HFD mice (*n* = 3), biological replicates. (**G**) Relative expression of TRIM32 protein in whole liver lysate of RCD and HFD mice (*n* = 5), biological replicates. (**H**) HepG2 cells were transfected with the TRIM32 plasmid. After 48 h, the cells were incubated with Biotin (2 mM), and surface INSR expression was analyzed using WB (*n* = 3, biological replicates). (**I**) Clustered Heat map showing differentially expressed ubiquitin ligases from the liver of healthy, early, and moderate human NAFLD patients (*n* = 216). (**J**) In these plots, we have shown individual transcripts per million (TPM) values as black dots across three independent groups of healthy, early, and moderate human NAFLD patients (*n* = 216). The minima in this plot is the lowest TPM value in the individual groups and maxima as the highest TPM value in the individual groups. The rectangle box represents the interquartile range (IQR), with quartile 1 (Q1) at the lower end of box and quartile 3 (Q3) at the upper end of the box. The black line inside the box (in between Q1 and Q3) represents the median value. The red colored dot in the center of each box represents mean (average) of all TPM values in the individual groups. Graphs were plotted in Graph pad prism8. The statistical significance was assessed by unpaired two-tailed t-test, and data has been expressed as Mean ± SEM. *p < 0.05, **p < 0.01, ***p < 0.001 and ****p < 0.0001. Data information: in (**F**), Relative mRNA expression of TRIM32 RCD vs. HFD (*p* = 0.0250), (**G**) Relative TRIM32/GAPDH (A.U) of RCD vs. HFD (*p* = 0.0079), (**H**) control vs. GFP-TRIM32(A.U) (*p* = 0.0143), (**J**) TRIM32 gene expression in Control vs. NAFLD mild (*p* < 0.0001), control vs NAFLD moderate (*p* < 0.0001), SREBF1gene expression Control vs NAFLD mild (*p* < 0.0001), control vs NAFLD moderate (*p* < 0.0001). Source data are available online for this figure.

Next, we examined the molecular mechanism of insulin receptor downregulation in the mouse primary hepatocytes. Here, we mimic the high-fat diet condition with hyperinsulinemia (HI). The use of HI conditions stems from various studies indicating that elevated insulin levels could potentially play a role in fostering insulin resistance through dietary means, including HFD (Choubey et al, 2020), and we check the levels of INSR in mouse primary hepatocytes. We found that HFD induced the degradation of INSR (Fig. 1I). To elucidate further the pathway involved in INSR downregulation, we treated primary hepatocytes with an inhibitor of the proteolytic pathway MG132. Notably, proteasomal inhibition through MG132 led to an increase in insulin receptor expression upon HI treatment (Fig. 2A). This result shows the involvement of the proteasomal pathway in INSR degradation.

Our data (Fig. 2A) also hinted that HI may influence INSR protein stability. To test this idea, endogenous INSR protein levels were measured by time after inhibition of protein synthesis with cycloheximide (CHX) (Choubey et al, 2020; Chattopadhyay et al, 2020). As shown in (Fig. 2B), levels of insulin receptors were reduced upon CHX treatment in the presence of HI. Together, these results clearly illustrate that HI reduced the protein stability of INSR. Since ubiquitination of the target protein precedes proteasomal degradation, we investigated the ubiquitination of insulin receptors in mouse primary hepatocytes using HI conditions. We also checked the ubiquitination level of INSR under high insulin conditions in mouse primary hepatocytes (Fig. 2C) and found increased ubiquitination of INSR in high insulin treatment. This result depicts that HI/HFD induces INSR ubiquitination and degradation through the proteasomal pathway.

Taking a cue from our data of HI/HFD-induced ubiquitination of insulin receptors to provoke their proteasomal degradation, we explored identifying the candidate (E3 ubiquitin ligase) that can act as a physiologically relevant repressor of insulin signaling.

E3 ubiquitin ligases are a large family of proteins that catalyze the ubiquitination of many proteins for degradation (Yang et al, 2016). RNA sequencing was performed from experimental mice livers to dissect the determinant that impairs cellular insulin action by degrading cell surface INSR in DIO mice. In RNA sequencing data, we found the expression of TRIM32 was upregulated in the livers of HFD mice (Fig. 2D,E, respectively), which was further confirmed by RT-PCR. Data suggested that among other E3 ubiquitin ligases (MRKN1, HUWE1, TRIM32, MARCH1 and TRIM72), HFD significantly induced only TRIM32 expression (Fig. EV2A–E, respectively). Next, we studied how hepatic TRIM32 regulates INSR expression in DIO mice. Like transcript level (Fig. 2F), we observed that the expression of TRIM32 protein was upregulated in HFD mice liver (Fig. 2G). To obtain insight into the physiological function of TRIM32, we examined TRIM32’s role in insulin receptor expression and its signaling axis using HepG2 cells and mouse primary hepatocytes. Membrane-bound proteins were extracted from HepG2 cells after TRIM32 overexpression, and INSR protein levels were quantified by Western blotting using the membrane protein fractions. The results showed that compared with un-transfected HepG2 cells, INSR protein levels had a significant reduction in the membrane fraction when TRIM32 was overexpressed (Fig. 2H). To determine the relevance of TRIM32 proteins in insulin resistance and NAFLD, we also examined the expressions of TRIM32 genes using publicly available hepatic transcriptome data for healthy and non-alcoholic fatty liver (NAFLD) patients (Fig. 2I). Interestingly, we found that expressions of TRIM32 and SREBF1 were significantly upregulated in the livers of NAFLD patients (Fig. 2J). Together, these results hinted that the HFD condition leads to increased expression of TRIM32, and INSR may be a potential TRIM32 substrate, whereas TRIM32 promotes INSR ubiquitination and its subsequent degradation. Therefore, next, we focused on TRIM32’s role in INSR signaling.

**Figure EV2:**

HFD induces TRIM32 expression to promote hepatic insulin receptor ubiquitination and degradation. (**A**–**E**) Quantitative representation of RT-PCR data for MRKN1, HUWE1, TRIM32, MARCH 1, and TRIM72 E3 ubiquitin ligases from RCD and HFD liver (*n* = 3), biological replicates. Graphs were plotted in Graph pad prism8. The statistical significance was assessed by unpaired two-tailed t-test, and the data has been expressed as Mean ± SEM. *p < 0.05, **p < 0.01, ***p < 0.001, and ****p < 0.0001. Data information: (**A**) RCD vs HFD (*p* = 0.1718), (**B**) RCD vs HFD (*p* = 0.3962), (**C**) RCD vs HFD (*p* = 0.0250), (**D**) RCD vs HFD (*p* = 0.4998), (**E**) RCD vs HFD (*p* = 0.0601). Source data are available online for this figure.

### TRIM32 induces ubiquitination and degradation of hepatic insulin receptors in mice with diet-induced obesity (DIO)

**Figure 3:**
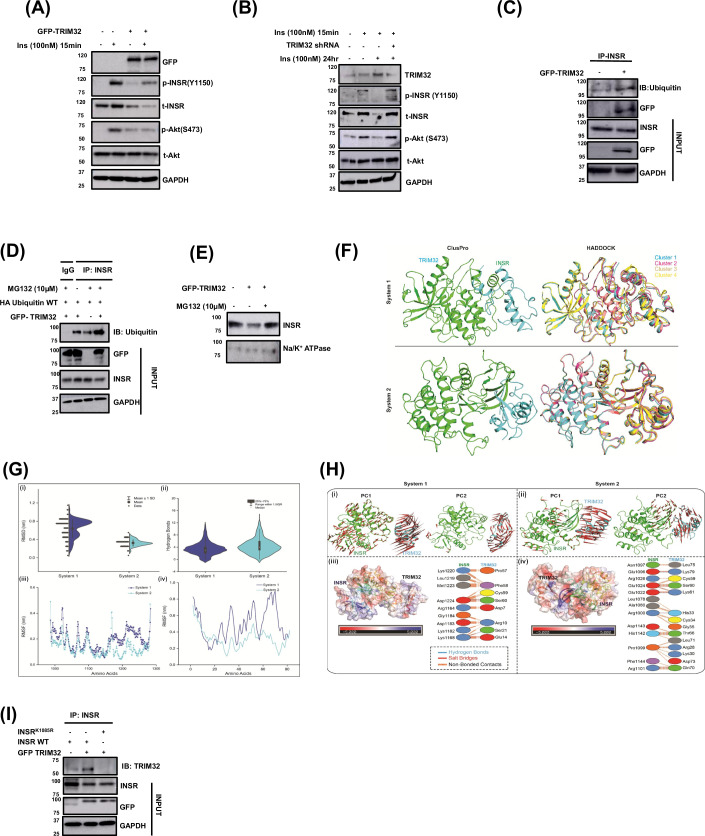
TRIM32 induces ubiquitination and degradation of hepatic insulin receptors in mice with diet-induced obesity (DIO). (**A**) TRIM32 was overexpressed in mouse primary hepatocytes and, after 48 h, treated with 100 nM insulin for the last 15 min to activate insulin signaling and accessed for pINSR(Y1150)/ tINSR and pAkt(S473)/tAkt using WB. (**B**) TRIM32 was knocked down in mouse primary hepatocytes. After 24 h of transfection, hepatocytes were treated with 100 nM insulin for the next 24 h. Hepatocytes were stimulated for 15 min with 100 nM insulin to access INSR and Akt phosphorylation using WB. (**C**) TRIM32 was overexpressed in HepG2 cells, and after 48 h of transfection, endogenous INSR was pooled down. The interaction of INSR and TRIM32, along with INSR ubiquitination, was assessed using WB. (**D**) TRIM32 and Ubiquitin-WT plasmids were overexpressed in HepG2 cells. After 24 h of transfection, 24 h of MG132 (10 μM) treatment were given, pooled down with INSR, and ubiquitination was accessed by WB. (**E**) TRIM32 was overexpressed in HepG2 cells and was treated with MG132(10 μM) for 24 h. Cells were incubated on ice with Biotin (2 mM) for the last 30 min, and surface INSR levels were analyzed using WB. (**F**) The plausible mode of interaction of INSR with TRIM32 as depicted by ClusPro and HADDOCK. Upper Panel Left: The top 1 ranked cluster with least energy having maximum number of structures obtained ClusPro. Upper Panel Right: Superimposed view of the top 1 ranked cluster snapshots obtained from HADDOCK. Lower Panel Left: The top 2 ranked cluster with least energy having maximum number of structures obtained ClusPro. Lower Panel Right: Superimposed view of the top 2 ranked cluster snapshots obtained from HADDOCK. (**G**) Intrinsic dynamics stability, inter-molecular hydrogen bonds and residual flexibility in INSR with TRIM32 complex systems over the time period of 100 ns. (**G**.i) Backbone RMSD of the two systems over the time period of 100 ns. (**G**.ii) The dynamics of inter-molecular hydrogen bonds in each frame of both the systems. (**G**.iii) The Cα-RMSF profile of the INSR protein with system 1 and 2 of INSR with TRIM32 complexes (**G**.iv) The Cα-RMSF profile of the TRIM32 protein with system 1 and 2 of INSR with TRIM32 complexes. (**H**) Global motions and the electrostatic surface potential maps of INSR with TRIM32 complex systems. (**H**.i) Porcupine plots of system 1 displaying the global movements of INSR-TRIM32 (INSR: Green; TRIM32: Cyan). (**H**.ii) The global movements of INSR-TRIM32 complex in system 2, displayed in porcupine plots (INSR: Green; TRIM32: Cyan). (**H**.iii) Distribution of surface charge of INSR-TRIM32 complex in system 1 and its intermolecular contact analysis. (**H**.iv) Electrostatic surface potential map of INSR-TRIM32 complex in system 2 and its subsequent intermolecular contacts. (**I**) Mouse primary hepatocytes were transfected with INSR-WT, INSRK1085R, and the TRIM32 plasmid. After 48 h, they were immunoprecipitated with the INSR antibody and accessed for TRIM32 using WB to check the interaction between INSR and TRIM32. Source data are available online for this figure.

To check the role of TRIM32 in INSR signaling, we overexpressed TRIM32 in mouse primary hepatocytes. We found that ectopic expression of TRIM32 was sufficient to blunt pINSR(Y1150), with consequent effects on Akt(Ser473) phosphorylation and significant reduction of soluble INSR fraction (Fig. 3A). Interestingly, this blunt phosphorylation of insulin receptors and Akt was rescued upon TRIM32 knockdown in HI condition in mouse primary hepatocytes (Fig. 3B). Collectively, these results suggest that TRIM32 plays a pivotal role in the regulation of insulin signaling activity by controlling cell surface INSR levels.

To find a correlation between TRIM32 and INSR levels, we wondered whether the two proteins are directly associated. We performed a co-immunoprecipitation assay to confirm the interaction of TRIM32 and INSR. The presence of TRIM32 in INSR immunoprecipitate was confirmed through immunoblotting, validating the interaction between TRIM32 and INSR (Fig. 3C). Moreover, in HepG2 cells with ectopically expressed GFP-TRIM32, we observed that TRIM32 triggers ubiquitination of INSR, as detected by immunoprecipitation using ubiquitin-specific antibodies (Fig. 3C). To validate further that TRIM32 leads to INSR ubiquitination, we performed an immunoprecipitation experiment in HepG2 cells after overexpressing HA-ubiquitin WT and GFP-TRIM32 plasmid in the presence and absence of MG132. As shown in (Fig. 3D), increased ubiquitination of INSR in the presence of ubiquitin, TRIM32, and MG132. Next, we performed a biotinylation experiment in HepG2 cells after GFP-TRIM32 overexpression and MG132 treatment. MG132 treatment significantly blocks the TRIM32-mediated ubiquitination and increases cell surface INSR levels (Fig. 3E). We further delineated the plausible domain-mediated specific interaction of TRIM32 with INSR through in silico protein–protein docking studies followed by validation through state-of-the-art all-atoms molecular dynamics simulation (Fig. 3F–H, respectively). The top two clusters (system 1 and system 2) obtained from HADDOCK after the refinement of the initial ClusPro-generated TRIM32-INSR complexes were selected for further analysis and optimization (Fig. 3F; Appendix Table S1). Top-ranked HADDOCK generated systems displayed two distinct sites mediated interaction, where in system 1, Lys1085, Arg1089, Arg1092, Asp1232, Asp1229, Gln1208, Gln1211, Asn1215, and Glu1216 of INSR formed crucial hydrogen bonds with Glu11, Glu11, Glu14, Arg55, Lys61, Met19, Phe58, His3, and Lys18 of TRIM32. However, system 2 showed a distinct network of hydrogen bonds and electrostatic interactions between INSR residues Arg1101, Glu1001, Glu1022, Glu1096, Arg1000, Ala1023, Glu1024, Arg1026, Gly1021, and TRIM32 N-terminal residues His3, Leu4, Asn5, Glu11, Arg28, Cys34, His36, Phe58, Cys59, Ser60, Lys61, Asn74, and Asp73 (Appendix Table S2). The structural stability of the top HADDOCK-generated TRIM32-INSR complexes (systems 1 and 2) was evaluated through 100 ns MD simulations. System 2 displayed higher stability, with lower backbone RMSD (0.32 ± 0.07 nm vs 0.64 ± 0.20 nm), greater compactness (lower radius of gyration and solvent accessible surface area), and more inter-molecular hydrogen bonds (5.07 ± 1.91 vs 4.67 ± 2.70 per frame) compared to system 1, indicating the most preferred state of molecular recognition (Appendix Fig. S1). Moreover, the degree of flexibility measured by Cα-RMSF profile also displayed lower flexibility for both TRIM32 and INSR in system 2 (Fig. 3G). Detailed post-simulation interaction analysis identified numerous stabilizing hydrogen bonds, salt bridges, and hydrophobic contacts between INSR and TRIM32 in system 2 as compared to system 1, indicating the most preferred conformational state (Fig. 3H; Appendix Fig. S2). From the docking study, we identified a possible site, i.e., K1085 on the INSR beta cytosolic subunit that can be a target for TRIM32 to bind, and we also generated an INSR mutant using SDM to mutate the site K1085R. These experiments revealed that the INSRs’ K1085 is required for the TRIM32- INSR interaction (Fig. 3I). Together, these results identify that the HFD leads to increased expression of TRIM32, INSR is a potential TRIM32 substrate, and TRIM32 induces ubiquitination and degradation of hepatic insulin receptors.

### HFD promotes SREBP-1c nuclear translocation to induce TRIM32 transcription

**Figure 4:**
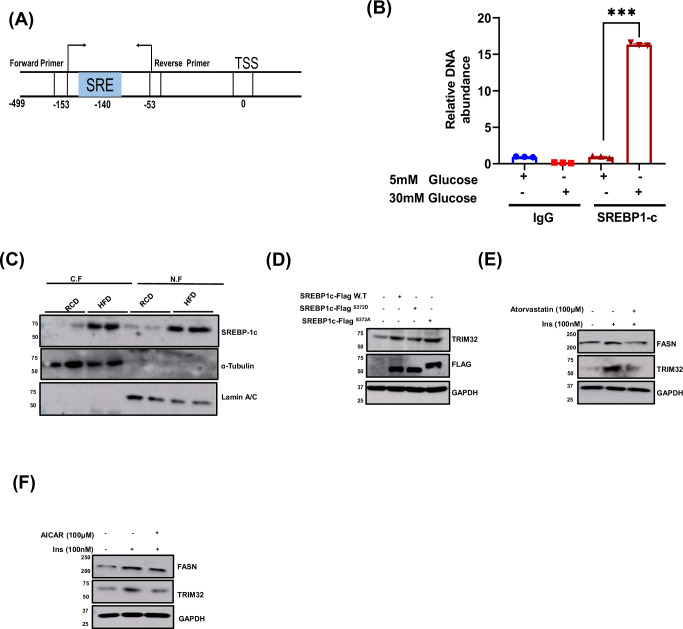
HFD promotes SREBP-1c nuclear translocation to induce TRIM32 transcription. (**A**) Schematic representation of SRE site on TRIM32 promoter. (**B**) SREBP-1c occupancy on TRIM32 promoter in HepG2 cells after high glucose stimulation (30 mM), (*n* = 3), biological replicates. (**C**) Qualitative representation of levels of SREBP-1c in the cytosolic and nuclear extract of RCD and HFD mice liver. (**D**) SREBP-1c WT, SREBP-1c S372A, and SREBP-1c S372D were overexpressed in mouse primary hepatocytes, and expression of TRIM32 was analyzed using WB. (**E**) Mouse hepatocytes were co-treated with Insulin (100 nM) and SREBP-1c inhibitor Atorvastatin (100 µM) for 24 h, and expression of TRIM32 and FASN was analyzed with WB. (**F**) Mouse hepatocytes were co-treated with Insulin (100 nM) and AMPK activator AICAR (100 µM) for 24 h, and expression of TRIM32 and FASN was analyzed with WB. Blots were quantified using ImageJ software, and values were plotted in GraphPad Prism 8. The statistical significance was assessed by one-way analysis of variance (ANOVA) post-Bonferroni. Data were expressed as Mean ± SEM. *p < 0.05, **p < 0.01, ***p < 0.001, and ****p < 0.0001. Data Information: In (**B**), 5 mM vs 30 mM glucose (*p* < 0.0001). Source data are available online for this figure.

Next, we wanted to investigate the regulation of TRIM32 expression in HFD-fed liver. Therefore, we checked the promoter of the TRIM32 gene using EPD (Eukaryotic Promoter Database) using JASPAR CORE (Transcription factor motif library). Analysis of the TRIM32 promoter revealed a putative SREBP-1c binding site SRE (Sterol regulatory element). We further confirmed the SREBP-1c binding site on the TRIM32 promoter through CHIP assay (Fig. 4A,B, respectively). It is known that SREBP-1c, upon activation, is translocated to the nucleus, enhancing the transcription of its target genes (Xu et al, 2013). Thus, we checked for cellular localization of SREBP-1c in the experimental mice groups. The nuclear pool from the HFD liver showed enhanced SREBP-1c accumulation in contrast to the RCD mice (Fig. 4C). To confirm that SREBP-1c regulates the TRIM32 expression, we ectopically overexpressed SREBP-1c in mouse primary hepatocytes, and data suggested that SREBP-1c induced TRIM32 expression (Fig. 4D). Our data predict that HFD induces SREBP-1c nuclear localization and promotes TRIM32 transcription, which mediates INSR ubiquitination and degradation. To further validate that SREBP-1c regulates TRIM32 expression, we treated mouse primary hepatocytes with a well-known SREBP-1c inhibitor, atorvastatin, in the presence of HI. HI-induced TRIM32 levels were blunted after atorvastatin treatment (Fig. 4E). These data hinted that HFD-induced TRIM32 expression by promoting the SREBP-1c nuclear localization.

As reported earlier, enhanced Ser372 phosphorylation on SREBP-1c by AMPK decreases the nuclear localization of SREBP-1c (Li et al, 2011). Here, we tested whether inactivated AMPK (HFD liver) further prompted hypophosphorylation (Ser372) of SREPB-1c and its nuclear translocation, leading to enhanced TRIM32 expression and hepatic insulin resistance. We designed two mutants of SREBP-1c, i.e., phosphomimic (S372D) and phospho-defective mutant (S372A). We found that upon wild-type SREBP-1c overexpression, TRIM32 protein levels were significantly elevated; further, with overexpression of the phospho-defective S372A mutant, the expression of TRIM32 was increased (Fig. 4D). These observations recommended that SREBP-1c Ser372 phosphorylation has a pivotal role in decreased nuclear localization of SREBP-1c and, in turn, regulates the expression of TRIM32. Further, the S372D mutant strongly diminished SREBP-1c transactivation and reduced TRIM32 expression.

To further check the specificity of TRIM32 inhibition by AMPK phosphorylation, we incorporated another well-known AMPK activator, i.e., AICAR. We found that AICAR-mediated AMPK activation downregulates TRIM32 expression (Fig. 4F). Taken together, phosphorylation and inactivation of SREBP-1c by AMPK may explain the beneficial effects on hepatic steatosis and hyperlipidemia with insulin resistance. Next, we checked whether depletion of TRIM32 in high-fat diet mice liver could rescue from insulin resistance and fatty liver.

### Depletion of TRIM32 in the liver of HFD mice increases insulin sensitivity and reverses fatty liver

**Figure 5:**
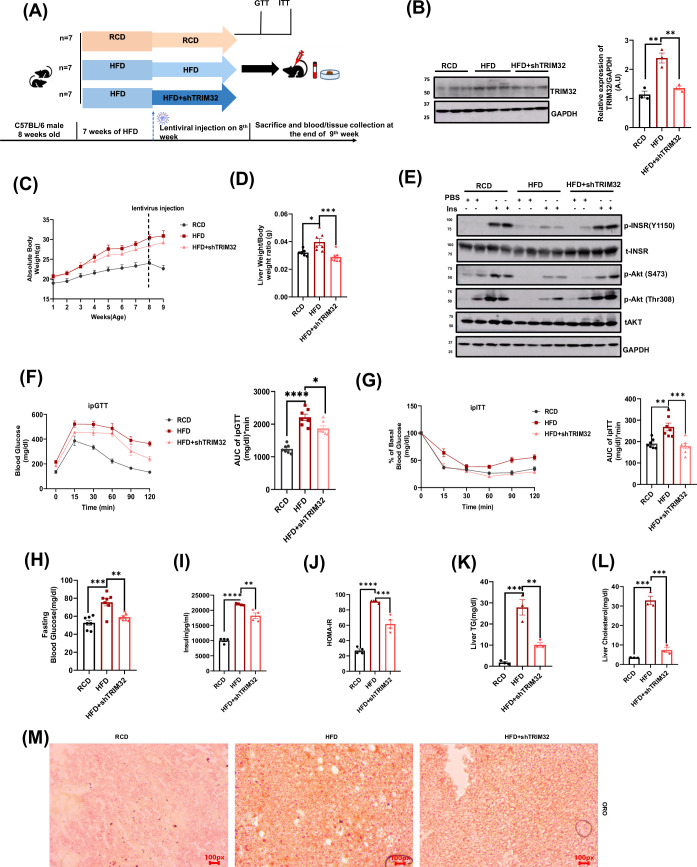
Depletion of TRIM32 in the liver of HFD mice increases insulin sensitivity and reverses fatty liver. (**A**) Schematic regimen of C57BL/6 male mice (*n* = 7 for each group). (**B**) Qualitative and quantitative representation of TRIM32 levels in RCD, HFD, and HFD+shTRIM32 group (*n* = 3), biological replicates. (**C**) Graphical representation of absolute change in body weight (*n* = 7). (**D**) The bar graph shows the relative change of liver to Body weight ratio (*n* = 7). (**E**) Qualitative representation of Western blot shows phosphorylation of INSR(Y1150) & Akt(S473 &Thr308), tAkt, respectively (*n* = 4), biological replicates. (**F**) Graphical representation of ipGTT data (*n* = 7). Mice were fasted for 6 h, 2 g/kg of B.W. glucose was injected intraperitoneally, and glucose level was measured at 0, 15, 30, 60, 90, and 120 min. AUC of ipGTT (*n* = 7). (**G**) Graphical representation of ipITT data (*n* = 7). Mice fasted for 6 h, 0.5 I.U/kg B.W. insulin was injected intraperitoneally, and blood glucose was measured at 0, 15, 30, 60, 90, and 120 min. AUC of ipITT (*n* = 7). (**H**) The bar graph shows relative fasting blood glucose levels (*n* = 7). (**I**) Relative Fasting insulin levels from mice serum after overnight fasting (*n* = 4), biological replicates. (**J**) The bar graph shows the relative HOMA-IR index (*n* = 4), biological replicates. (**K**, **L**) The bar graph shows triglyceride and cholesterol levels in the liver of mice (*n* = 4), biological replicates. (**M**) ORO images from RCD, HFD, and HFD+shTRIM32 mice liver sections. Images were taken at 40x. Blots were quantified using ImageJ software, and values were plotted in GraphPad Prism 8. The statistical significance was assessed by one-way analysis of variance (ANOVA) with Bonferroni. Data were expressed as Mean ± SEM. *p < 0.05, **p < 0.01, ***p < 0.001, and ****p < 0.0001. Data information: In (**B**), RCD vs HFD (*p* = 0.0013) and HFD vs HFD+shTRIM32 (*p* = 0.0036), (**D**) RCD vs HFD (*p* = 0.0149) and HFD vs HFD+shTRIM32 (*p* = 0.0008), (**F**) AUC of ipGTT RCD vs HFD (*p* = 0.0001) and HFD vs HFD+shTRIM32 (*P* = 0.0179), (**G**) AUC ipITT RCD vs HFD (*p* = 0.020) and HFD vs HFD+shTRIM32 (*p* = 0.0009), (**H**) Insulin RCD vs HFD (*p* = 0.0001) and HFD vs HFD+shTRIM32 (*p* = 0.0048), (**I**) Insulin RCD vs HFD (*p* = 0.0001) and HFD vs HFD+shTRIM32 (*p* = 0.0030), (**J**) HOMA-IR of RCD vs HFD (*p* < 0.0001) and HFD vs HFD+shTRIM32 (*p* = 0.0004), (**K**) Liver TG of RCD vs HFD (*p* = 0.0005) and HFD vs HFD+shTRIM32 (*p* = 0.0039), (**L**) Liver Cholesterol of RCD vs HFD (*p* = 0.0001) and HFD vs HFD+shTRIM32 (*p* = 0.0001). Source data are available online for this figure.

**Figure EV3:**
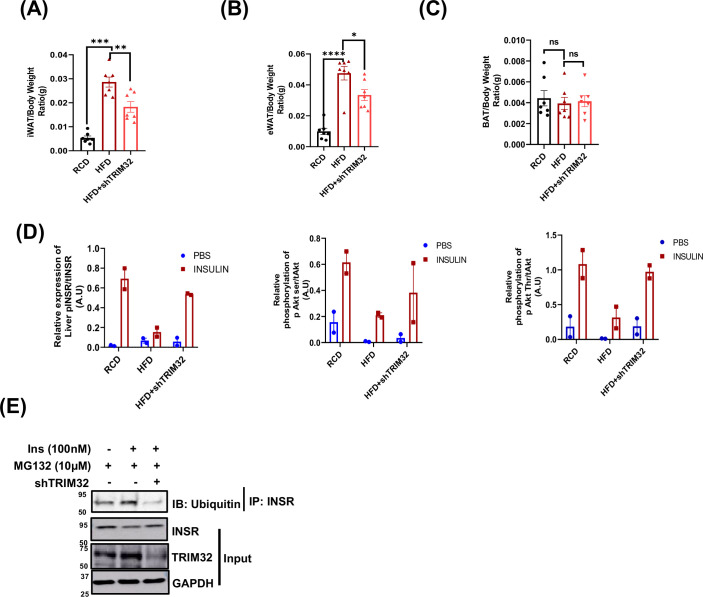
Depletion of TRIM32 in the liver of HFD mice increases insulin sensitivity and reverses fatty liver. (**A**–**C**) The bar graph shows the relative change of iWAT, eWAT, and BAT to body weight ratio (*n* = 7). (**D**) Quantitative representation of Western blots showing phosphorylation of INSR(Y1150) (left), Akt(S473) (middle), and Akt(Thr308) (right) (*n* = 3), biological replicates. (**E**) Mouse primary hepatocytes were transfected with the shTRIM32 plasmid and, after 24 h, co-treated with Insulin (100 nM) and MG132 (10 µM) for the next 24 h. Immunoprecipitation was performed using the INSR antibody and checked for INSR ubiquitination using WB. Blots were quantified using ImageJ software, and values were plotted in GraphPad Prism 8. The statistical significance was assessed by one-way analysis of variance (ANOVA) with Bonferroni. Data were expressed as Mean ± SEM. *p < 0.05, **p < 0.01, ***p < 0.001 and ****p < 0.0001 Data information in (**A**) iWAT by B.W. ratio of RCD vs. HFD (*p* < 0.0001) and HFD vs. HFDshTRIM32 (*p* = 0.0025), (**B**) eWAT by B.W. ratio of RCD vs. HFD (*p* < 0.0001) and HFD vs. HFDshTRIM32 (*p* = 0.0287), (**C**) BAT by B.W. ratio of RCD vs HFD (*p* = 0.099) and HFD vs HFDshTRIM32 (*p* = 0.0999). Source data are available online for this figure.

Next, to examine whether TRIM32 is sufficient to impair insulin action in vivo, we used a lentiviral approach to ectopically knock down TRIM32 in the liver of HFD-induced DIO mice. To this end, 8-week-old male C57BL/6 mice were fed with HFD for 9 weeks. The TRIM32 gene was knocked down in the liver via lentivirus expressing TRIM32-specific shRNA (Lv-shTRIM32) at the end of 7th week of the high-fat diet (Fig. 5A). After 1 week of shRNA injection ipGTT, ipITT was performed and mice were sacrificed at the end of 9th week. Knockdown was specific as reflected on immunoblot in the liver lysates (Fig. 5B). Mice receiving shRNA for TRIM32 did not significantly differ in body weight and adiposity but gained less fat mass than the HFD group (Fig. 5C, Fig. EV3A–C, respectively). Figure 5D, revealed a reduced liver-to-body weight ratio and improved hepatic function in HFD-fed obese mice by down-regulating TRIM32. To uncover TRIM32’s role in modulating the hepatic insulin signaling pathway, we examined the expression patterns of pAkt and pINSR, which serve as indicators for INSR receptor signaling, across RCD, HFD, and HFD+shTRIM32 mouse groups. Our findings revealed that HFD decreased insulin receptor signaling, whereas the introduction of TRIM32-shRNA in HFD mice notably boosted insulin signaling (Figs. 5E and EV3D).

In the intraperitoneal glucose tolerance tests conducted on high-fat diet (HFD) fed mice, the suppression of TRIM32 resulted in enhanced glucose tolerance, as depicted (Fig. 5F). In addition, improved systemic insulin sensitivity was evident in both intraperitoneal glucose tolerance tests (ipGTT) and intraperitoneal insulin tolerance tests (ipITT), as illustrated in (Fig. 5F,G, respectively). Data from mouse primary hepatocytes showed that high insulin-mediated INSR ubiquitination was rescued upon TRIM32 knockdown (Fig. EV3E). On a similar line, HFD-fed mice with shTRIM32 injection have lower fasting glucose (Fig. 5H), reduced circulating insulin (Fig. 5I) and improved insulin sensitivity (HOMA-IR) index (Fig. 5J). Altogether, our findings present in vivo evidence supporting TRIM32’s role in the negative regulation of liver insulin receptor signaling in mice with HFD-induced insulin resistance. Moreover, the accumulation of liver TG and cholesterol levels decreased in the shTRIM32 group of HFD mice (Fig. 5K,L, respectively). We found that the shTRIM32 group has lower lipid accumulation, as depicted by ORO images (Fig. 5M). This shows that TRIM32 downregulation in HFD obese mice liver can rescue the mice from developing fatty liver and improve insulin sensitivity. It turned out that TRIM32 is one of the modulators of the hepatic insulin signaling axis, and our findings also illustrate in vivo evidence for negative regulation of liver insulin receptor signaling by TRIM32 in HFD-induced insulin-resistant mice.

## Discussion

Utilizing a holistic approach encompassing in vitro and in vivo experiments, we identified the E3 ubiquitin ligase TRIM32 as a critical regulator of INSR degradation during the development of HFD-induced insulin-resistance in mice. We found that TRIM32 degrades insulin receptors and impairs insulin sensitivity. Analysis of mouse liver revealed an inappropriate surge in TRIM32 expression among mice subjected to an HFD. These findings suggest a direct sequence wherein the HFD induces nuclear translocation of SREBP-1c which potentially instigates TRIM32 expression. We also checked TRIM32 expression from an online available RNA sequencing dataset of the human liver of healthy and NAFLD patients (Govaere et al, 2020). The data shows that NAFLD patients have increased TRIM32 gene expression and its regulatory transcription factor, the SREBF1 gene. Our finding shows that HFD induces nuclear translocation of SREBP-1c (Linden et al, 2018; Horton et al, 2002), further instigating TRIM32 expression. This, in turn, governs INSR stability via direct ubiquitination, consequently decreasing insulin receptor expression in mice’s liver. Previous studies have shown an inverse correlation between AMPK and SREBP-1c activities in hepatocytes and the liver (Li et al, 2011). HFD hampers AMPK activity, which ultimately leads to increased SREBP-1c nuclear translocation. It is largely known how AMPK regulates SREBP-1c activity in controlling lipid homeostasis. However, how AMPK regulates insulin receptor signaling through SREBP-1c is largely unknown, especially in the insulin-resistant state.

Our study utilized two SREBP-1c constructs with a mutation on their Ser372 phosphorylation site by AMPK, i.e., Flag-SREBP-1c^S372A^ & Flag-SREBP-1c^S372D^. Our results indicated that the phospho-defective mutant of SREBP-1c, i.e., Flag-SREBP-1c S372A, translocates inside the nucleus and increases TRIM32 expression. We also incorporated SREBP-1c inhibitor Atorvastatin (Zhang et al, 2020) and AMPK activator (AICAR) (Tomita et al, 2005; Liu et al, 2015) to establish that SREBP-1c regulates hepatic TRIM32 levels. Our studies reinforce AMPK as a direct upstream kinase that phosphorylates SREBP-1c to inhibit its nuclear translocation and transcriptional activity, ultimately suppressing hepatocyte lipogenesis and INSR degradation implicated in the rescuing from NAFLD and insulin resistance. Moreover, these data also explain why AMPK activators exert a beneficial effect on obesity-induced aberrant triglyceride metabolism, hepatic steatosis, and as well as insulin resistance.

Altered INSR levels and kinase activity have been documented in individuals with obesity and NAFLD (Wang et al, 2019; Tavare et al, 1988), potentially linked to the downregulation of INSR induced by high-fat diet (HFD) conditions. Dysregulated functions of E3 ubiquitin ligases have been linked to many diseases, including T2DM and NAFLD, making them potential targets for drug development. The potential of developing therapeutic approaches that target specific E3 ubiquitin ligases is promising. However, a significant gap in this area is the lack of understanding regarding the mechanisms that control the expression and activity of E3 ubiquitin ligases. This study identifies the E3 ubiquitin ligase TRIM32 as a critical factor in the degradation of the INSR, leading to a reduction of membrane-associated INSR protein levels and insulin resistance.

In conclusion, our study showed that obesity induced by a high-fat diet triggers two significant responses: first, it inhibits the AMPK-dependent Ser372 phosphorylation of SREBP-1c, consequently enhancing the transcriptional activity of TRIM32. This, in turn, leads to insulin receptor degradation and promotes insulin resistance in obese mice. Second, nuclear-localized SREBP-1c also boosts hepatic DNL and the accumulation of intrahepatic lipids (Horton et al, 2002). Our results support a prevailing role of hepatic insulin receptors over that of other tissues (Kido et al, 2000) in the pathology of obesity-induced insulin resistance-driven metabolic disease. Based on these findings, we propose that the TRIM32-INSR to be a potential site for pharmacological interventions as an effective therapy in mitigating obesity-induced hepatic insulin resistance, associated fatty liver disease, and dyslipidemia.

## Methods

**Table:** Reagents and tools table

Reagent/Resource	Reference or Source	Identifier or Catalog Number
**Experimental models**		
C57BL/6J (*M. musculus*)	IISER Mohali, Chandigarh, India	NA
HepG2	ATCC	HB-8065
293T	ATCC	CRL-11268
**Recombinant DNA**		
pEGFP-N1_hTRIM32	Addgene	69541
HA-UBIQUITIN WT	Addgene	17608
pcDNA3.1-2xFLAG-SREBP-1c	Addgene	26802
GFP-hIR	Addgene	24049
pLKO.1 TRIM32 shRNA plasmid	Merck	TRCN0000040829
**Antibodies**		
pAKT S473	CST	4058S
pAKT T308	CST	4056
tAKT	CST	9272s
pAMPKα	CST	2535s
tAMPKα	CST	2535
pACC	CST	3661
t-ACC	CST	3676
GAPDH	CST	D16h11(5174)
*α*-tubulin	CST	2144
Phospho-IGF-I Receptor β (Tyr1135/1136)/Insulin Receptor β (Tyr1150/1151)	CST	3024
INSR	CST	3020s
GFP	Novus	NB600-308
FASn	ABclonal	A0461
TRIM32	ABclonal	A7079
Lamin A/C	CST	4777s
Mouse-Anti-flag	Sigma	F1804
SREBP1-c	Novus	NB100-2215
Na/K^+^ ATPase	CST	3010S
**Oligonucleotides and other sequence-based reagents**		
**Primer ID**		**Sequence**
mMARCHF7 F. P	*M. musculus*	TCTTGGAGGCATAGTCAAGTTC
mMARCHF7 R. P	*M. musculus*	GACTCTCTTCTCCTGTCCAAATC
mHUWE1F.P	*M. musculus*	CACTGTCCTCCTCTCTCTATGT
mHUWE1 R.P	*M. musculus*	ACCTGCCTTCTCATCCTTTG
mMKRN1F.P	*M. musculus*	CACTGGTCTGGCTGAAGATAAG
mMKRN1R.P	*M. musculus*	CTGATGGCATCTAGGTAGGAATG
m18srRNA F. P	*M. musculus*	GCAATTATTCCCCATGAACG
m18srRNA R. P	*M. musculus*	GGCCTCACTAAACCATCCAA
h IR_K2R_F. P	*Homo sapiens*	AGTGGTGTCCAGGGGCCAGCCCA
h IR_K2R_R. P	*Homo sapiens*	CCCAGGAGGCGCACCACG
mTRIM72. F. P	*M. musculus*	CTGGACCGTGAAGCAGAAA
mTRIM72. R. P	*M. musculus*	GCCTCAGTTGCTCTAGGTAAG
**Chemicals, Enzymes, and other reagents**		
Bouin’s solution	Sigma	MFCD00146169
I Script cDNA synthesis kit	Bio-Rad	170881
iTaq Universal SYBR Green Supermix	Bio-Rad	1725121
Insulin	Sigma	I0516
Gateway cloning system	Invitrogen	V49320
Lipofectamine 3000	Invitrogen	L3000015
Lipofectamine RNAimax	Invitrogen	13778150
BCA assay	Thermo Scientific	23227
Nuclear and cytoplasmic extraction kit	Thermo	78833
ORO	Sigma	O0625
Fluoroshield™ with DAPI	Sigma	F6057
Q5^®^ Site-Directed Mutagenesis Kit	NEB	E0554S
Amicon Ultra-15 centrifugal filter	Merck	UFC901008
EZ-Link™ Sulfo-NHS-SS-Biotin	Invitrogen	21331
Protease inhibitor cocktail	Sigma	P8340
Nitrocellulose membrane, 0.2 µm	Bio-Rad	1620112
Collagen1, from Rat Tail	Thermo	A1048301
Collagenase from Clostridium histolyticum	Sigma	C0130
AICAR	Sigma	A9978
**Software**		
Graph Pad Prism 8		
ImageJ		
**Other**	Company	Catalog no
Gel DOC	Amersham	AI600
BIOPLEX	BIORAD	

### In vivo studies

#### Animals

We obtained male C57BL/6 mice (8 weeks old) from the Animal House of IISER Mohali, India, and allowed them to acclimatize for 1 week at 25 °C with 50–60% humidity, under a 12:12-h light/dark cycle, and provided free access to food and water. All experimental procedures received approval (IIT/IAEC/2023/002) from the Institute Animal Ethics Committee at the Indian Institute of Technology Mandi, the Ministry of Environment and Forest, and the Government of India. Further, the mice were divided into two groups comprising ten animals. Group 1 received a regular chow diet (~14% calories from fat, 20% protein, 14.725% carbohydrate) (5L79 Lab diet). Group 2, known as HFD mice, were fed a high-fat diet (60% calories from fat, 20% protein, 20% carbohydrate, and an energy density of 5.21 kcal/g) (Research Diet, D12492 (Van Heek et al, 1997)) for 8 weeks. The ipGTT (intraperitoneal glucose tolerance test) and ipITT (insulin tolerance test) were conducted after 8 weeks of HFD. Before these tests, the mice underwent a 6-h fast with free access to water, and basal glucose levels were measured before injection. For ipGTT, mice received an injection of 2 g/kg body weight glucose, while for ipITT, insulin (0.50 I.U/kg B.W.) was administered via intraperitoneal injection. Blood glucose levels were measured at specified time points: 15, 30, 60, 90, and 120 min post-injection. ipPST (intraperitoneal pyruvate tolerance test) involved an overnight fast with free access to water, followed by an injection of 100 mg/kg body weight of Na/Pyruvate (0.9% saline). Basal blood glucose levels were measured at zero time points. Glucose levels were measured 15, 30, 60, 90, and 120 min post-injection. At the end of the 8th week, following an overnight fast, fasting blood glucose was measured the following day, and the serum was collected. After sacrifice, serum and tissues were collected and snap-frozen for protein and RNA isolation. Tissues for histopathological examination were fixed in Bouin’s fixative overnight, followed by PFA for 4 h, and subsequently washed in 70% ethanol and 30% sucrose for preservation. For each in vivo experiment, mice cohorts were randomized, followed by the respective lentivirus treatment for shTRIM32 via intravenous injections.

#### Preparation of lentivirus

For TRIM32 knockdown, 293T cells were transfected with pLKO.1 vector containing the shRNA sequence (CCCAAGTTTGTCACCTGTGAT) (Sigma) for mouse TRIM32 along with two packaging vectors-psPAX2 and pMD2.G (Addgene). The lentiviral particles were generated and further purified and concentrated using an Amicon Ultra-15 centrifugal filter (UFC905008), and ~1 × 10^12^ pfu/mice were administered via tail vein injection.

### In vitro study

#### Cell culture and treatment

HEK293T and HepG2 (human hepatocellular carcinoma) cells were cultured in Dulbecco’s modified Eagle’s medium (DMEM High Glucose; Invitrogen) supplemented with 3.7 g/L NaHCO_3_, 10% fetal bovine serum (Invitrogen, sourced from the US), 100 U/ml penicillin, and 100 μg/ml streptomycin in a 5% CO_2_ incubator at 37 °C. Cells were treated with 100 nM insulin for 24 h to induce hyperinsulinemia, and for acute stimulation of Akt signaling, 15-min insulin (100 nM) treatment was employed. For the inhibition of protein translation, CHX treatment was implemented at 100 µg/ml for time intervals of 0, 2, 4, and 6 h. MG132 at a concentration of 10 µM was used for all experiments.

#### Primary hepatocyte culture

Briefly, 8-week-old mice’s livers were perfused with a 30 ml calcium-free HBSS buffer (pH 7.4) containing 1 mM EDTA via the vena cava at a 3 ml/min speed. At this point, the liver will turn pale, and then, HBSS with Ca^2+^ containing 0.5 mg/ml collagenase is perfused into the liver. Noticing the crack on the surface of the liver, perfusion was stopped immediately, and the liver was excised into ice-cold FBS-free William’s-E media. Cells from digested livers were teased and filtered through a 100 µm cell strainer and centrifuged at 20 × *g* for 3 min at 4 °C. The pellets were washed with FBS-free William’s-E media twice. The cells were cultured in William’s-E media without FBS on collagen-coated plates. The media was replaced with complete William’s-E media containing 10% FBS supplemented with 1% pen-strep after 4–6 h. AMPK activator, AICAR, was used at a dose of 100 µM. Atorvastatin at a concentration of 100 µM was used.

#### Transfection protocol

Transfections were performed using Mouse primary hepatocytes and HepG2 cells. All overexpression experiments were carried out using Lipofectamine 3000 (Invitrogen). pEGFP-N1_hTRIM32, GFP-hIR, and pcDNA3.1-2xFLAG-SREBP-1c plasmids were used, and transfection was done according to the manufacturer protocol (Lipofectamine 3000, Invitrogen). Media was changed after 4 h of transfection with complete media (DMEM).

#### Western blotting

Cells or tissues were lysed in RIPA lysis buffer containing 1x protease (Sigma) and phosphatase inhibitor cocktail (CST). Cells were incubated on ice for 45 min with vortexing in between and then spun at 15,000 rpm for 15 min, respectively. The protein was further quantified using a BCA reagent kit (Thermo Scientific #23227). Protein was loaded on SDS-PAGE and transferred to the PVDF membrane (0.2 µm). After transfer, the membrane with proteins was washed with 1x TBST for 5 min and then blocked with 5% milk for 1 h at room temperature. The membrane with the protein of interest was incubated overnight with a primary antibody in a dilution of 1:2000 (5% BSA in 1x TBST). After overnight incubation, the membrane was washed with 1x TBST thrice for 10 min each. The membrane was then incubated with HRP-conjugated secondary antibody for 2 hr at room temperature. The blot was developed using ECL in Amersham gel doc-AI600.

#### Co-immunoprecipitation

HepG2 cells were used for immunoprecipitation experiments to determine the interaction between TRIM32 and INSR. pEGFP-N1_hTRIM32 was overexpressed using lipofectamine 3000 (Invitrogen) and after 48 h of transfection cells were lysed in NP-40 Lysis Buffer (NaCl 100 mM, HEPES 30 mM, NP-40 0.5%) of pH 7.4 with protease inhibitor. Protein was isolated by centrifugation at 15,000 rpm for 15 min, after 45 min of incubation on ice with 10 s of gentle vortexing. Protein was estimated with a BCA kit (Thermo Scientific). Dynabeads^TM^ were washed with PBS, and 800 µg protein was incubated with beads and Insulin receptor antibody. Beads were incubated with protein and antibody overnight, and the next day, the beads were washed with a washing buffer (Tris 50 mM, NaCl 100 mM) pH 7.4, three times. Protein was eluted in the Laemmle buffer with 5% BME and preceded for western blotting.

#### Nuclear cytosolic

SREBP-1c nuclear translocation in liver tissue of RCD, HFD mice was assessed using the nuclear-cytosolic extraction kit (Thermo) followed by manufacturer protocol. 15 mg of liver tissue was used. Nuclear and cytosolic fractions were analyzed using western blotting.

#### Cell surface biotinylation assay

Cells were used for the cell surface biotinylation experiment. Cells were seeded in a 60 mm culture dish, and after treatment time, cells were washed with 1x PBS. The 10 mM original stock solution of EZ-Link™ Sulfo-NHS-Biotin (Sigma) was prepared in PBS, and a 2 mM final treatment was given. Cells were incubated at 4 °C with gentle shaking for 30 min. Cells were washed three times with 100 mM Glycine (in PBS) to remove excess biotin. Cells were lysed using RIPA lysis buffer, and further protein extraction was performed. Protein was estimated with a BCA kit (Thermo). Protein was incubated overnight with Agarose beads. The next day, Beads were washed with PBS 3 times in Laemmle buffer and loaded for SDS-PAGE.

#### Immunohistochemistry

Bouin’s fixed liver tissue was embedded in paraffin, and 5 µm sections were made, deparaffinized, rehydrated, and washed with 1x PBS. Microwaving slides performed antigen retrieval for 15 min with sodium citrate buffer (pH 6). Slides were washed and permeabilized with 0.1% Triton-X in PBS. The tissue was blocked with 2% Neonatal horse serum for 1 h and incubated with 1:300 dilution F4/80 (CST) overnight at 4 °C. The next day, the Slides were washed with PBST, incubated with 1:1000 dilution secondary antibody (Alexa-647) for 2 h, and mounted with DAPI mounting media. Imaging was done using a confocal microscope (Nikon).

#### Staining protocol

Cells were plated on the coverslip, and the required treatment was given. For Bodipy, cells were washed with PBS and then fixed with a 3% PFA solution at room temperature for 15 min. Cells were washed three times. 2 µM Bodipy was the final concentration per well and incubated at 37 °C for 30 min. Cells were washed with PBS 3 times. Coverslips were dried and mounted with DAPI mounting media (Himedia). Images were captured using Nikon confocal microscopy.

#### Histopathological analysis

Frozen liver tissue samples during the sacrifice (Bouin’s solution, Sigma MFCD00146169) were used for paraffin sections. Hematoxylin-eosin (HE) staining was done for liver and adipose tissue. ORO staining was done to evaluate the lipid accumulation in the liver. Sirius red staining was done to assess hepatic fibrosis.

#### Serum insulin level measurement

Serum insulin levels were measured using a Bio-Plex ProTM Diabetes Reagent kit (Cat# 171F7001M) on a Bio-Plex-200 (Bio-Rad) following the manufacturer’s instructions. HOMA-IR levels were calculated from fasting insulin levels and fasting plasma glucose levels using the formula—(Fasting insulin × Fasting plasma glucose levels)/405.

#### Gene expression level studies

Total RNA from liver tissues and cells was extracted using RNA XpressTM reagent (Himedia MB601) following the procedure recommended by the manufacturer. Per the manufacturer’s guidelines, 1 μg of total RNA was processed for cDNA (iScript cDNA synthesis kit, Bio-Rad 170881). Quantitative PCR was then performed using the SYBR mix following iQ SYBR Green Supermix (Bio-Rad 1725121). Gene expression fold analyses were carried out using 2^−ΔΔCT^ expression where ΔΔCT represents the relative change difference among the control and treated groups post normalization with 18s rRNA (mouse).

#### Protein–protein docking

In this study, the X-ray crystallographic structure of TRIM32 RING Domain (PDB ID:5FEY) and INSR subunit beta(3BU3) were obtained from protein data bank (PDB) and subsequently utilized for protein:protein docking ClusPro (Kozakov et al, 2017) and HADDOCK (Yan et al, 2020) tools were used and followed by all-atoms molecular dynamics simulations to infer the plausible mode of interaction of TRIM32 with INSR. The protein preparation wizard of Maestro 12.8 (Schrödinger, LLC, New York, 2021-22) was used to prepare both the proteins by optimization of hydrogen bonds, and the addition of missing hydrogen atoms was carried out with PROPKA 3.0 at pH 7.0. The optimized structures of TRIM32 and INSR were submitted to ClusPro for protein–protein docking with default parameters. The top two cluster representatives (with a maximum number of structures having higher weighted score) obtained from ClusPro were subjected to further refinement using High Ambiguity Driven protein–protein Docking v2.4-2022.08 (HADDOCK), where interacting residue pairs of the top complex (obtained from ClusPro) were supplied as active site residue for flexible docking of biomolecular complexes, The top two clusters with higher HADDOCK Z-score (negative) were retained further for non-bonded contact analysis and optimization using all-atoms MD simulations.

#### All-atoms molecular dynamics simulations

All-atom molecular dynamics simulations of systems of the top two ranked complexes of TRIM32-INSR obtained from HADDOCK using CHARMM36m forcefield (Huang et al, 2016) in TIP3P water models using a rectangular box in GROMACS (Abraham et al, 2015). Both the systems were electro-neutralized with addition of an appropriate amount of NaCl (0.15 M) counterion. Electro-neutralization was followed by energy minimization to remove bad contacts using steepest descent method with a total of 5000 steps. Subsequently, equilibration was performed using NVT and NPT ensemble with constant volume and constant pressure to gradually bring the systems to the target temperature of 300 K and pressure of 1 atm for 5 ns and 10 ns, respectively. For NVT, Nose-Hoover method was employed to maintain temperature of the system while, for NPT, Parinello–Rahman algorithm was employed to maintain the pressure. Finally, a production MD simulation of 100 ns was conducted using Leap-Frog integrator (Hockney et al, 1974) to understand the structural-stability and dynamics of TRIM32-INSR systems. The non-bonded interactions were treated using Verlet cut-off scheme, where, the particle mesh Ewald (PME) method was employed to treat long-range electrostatic interactions. The short-range electrostatic and van der Waals interactions were calculated with a cut-off of 12 Å. Bonds concerning hydrogen atoms were constrained using linear-constraint-solving (LINCS) algorithm (Hess et al, 1997). The trajectories were analyzed using the GROMACS built-in programs. The visualization programs VMD and PyMOL were used to examine the molecular dynamics trajectories and analyze interactions within TRIM32-INSR systems. The top-ranked cluster representatives using GROMOS-based clustering analysis (with an RMSD cut-off of 0.2 nm) of the MD trajectory was used to compute the electrostatic surface potential of the TRIM32-INSR protein–protein complexes using the APBS plug-in tool of PyMOL with a bulk solvent radius of 1.4 Å and a dielectric constant of 78. The electrostatic positive and negative surface patches in both the complexes were observed using a contour (kT/e) value of 1.

#### Isolation of hepatic TG and cholesterol

Frozen liver tissue (50 mg) was used and homogenized in 450 µl of ice-cold PBS. 200 μl of homogenized lysate was transferred to a fresh tube containing 1200 μl of methanol:chloroform in the ratio (2:1) and mixed with vigorous shaking. 100 μl of PBS was added into the tube and mixed vigorously. The mixture was centrifuged at 4200 rpm for 10 min at 4 °C. 200 μl of the organic phase was then transferred into a fresh tube and dried until fully evaporated using a speed vacuum. 200 μl of 1% Triton X-100 in ethanol was used to dissolve the dried lipid pellet. Cholesterol and TG content were measured using Triglycerides (MAK266, Sigma-Aldrich) and Cholesterol (AB65359, Abcam) estimation kits (Rawat et al, 2023; Dogra et al, 2022).

#### Biochemical parameter analysis

Blood was collected from the heart during the mice’s sacrifice. The serum was isolated after centrifugation at 1000 × *g* for 5 min. Serum ALT (MAK052, Sigma-Aldrich), AST (MAK055, Sigma-Aldrich), and TG (MAK266, Sigma-Aldrich) were measured according to the manufacturer protocol.

#### RNA sequencing

The total RNA (500 ng) was used to prepare libraries for RNA sequencing using QIAseq Stranded mRNA Select Kit (96) (Cat# 180775) as per the manufacturer’s protocol. The RNA was mRNA enriched, converted to cDNA, fragmented, end-repaired, adenylated, and ligated with index adapters to prepare into the library according to the kit protocol (QIAGEN Cat# 180775). The library was purified using QIAseq beads (provided with a kit). The library was subjected to 12-PCR cycles of amplification using primers provided in the kit (QIAGEN Cat# 180775). The PCR products (final amplified library) were purified using QIAseq beads. The quantification and size distribution of the prepared library was accomplished using a Qubit fluorometer and Agilent Bioanalyzer (Agilent Technologies) following the manufacturer’s instructions.

#### RNA sequencing data analysis

CLC Genomics WB has been used (21.0.5) for QC-check of Fast Q, and data have been quality trimmed based upon quality scores (limit = 0.05), ambiguous nucleotides (Max = 2), Homopolymers from 3’ end for all bases and removed 15 bases from 5’ end of each read. Trimmed data have been aligned with the reference Genome annotated with genes and transcripts using the reference genome of Mus_musculus_ensembl_v80_Sequence provided with mRNA (mus_musculus_refseq_GRCm38.p6_mRNA) and gene (mus_musculus_refseq_GRCm38. p6_Genes) information (known as reference-based assembly) with parameters (Mismatch cost = 2, Insertion cost = 3, Deletion cost = 3, Length fraction = 0.8, Similarity fraction = 0.8, Strand specific = Both, Maximum number of hits for a read = 10, Count paired reads as two = No, Expression value = Unique counts).

#### Differential gene expression analysis

Raw values (unique read counts) for Gene Expression (GE) have been taken forward to determine differentially expressed genes using the DESeq2 package in R (version 4.2.1). Upregulated and downregulated genes were identified at cut-off with adjusted-value = 0.5 and Fold-Change > abs (2). Further analysis of common and unique up- and down-regulated genes was carried out using a Venn diagram to obtain standard and unique gene sets of differentially expressed genes.

### Biological interpretation of RNA sequencing data

#### Gene ontology (GO) annotation

Enrichment analysis has been performed on Meta Scape (http://metascape.org) for differentially expressed genes having fold change abs (>2) & *p*-value ≤ 0.05. Enrichment of Biological Processes, Molecular Functions, Cellular components, and KEGG Pathways was carried out at cutoff values of Min Overlap = 2, *P*-Value Cutoff = 0.05, and Min Enrichment = 1.5.

#### Utilization of online available RNA sequencing dataset

RNA sequencing data analysis was performed using a publicly available dataset from the Gene Expression Omnibus (GEO) database with Accession no. GSE135251. This dataset reports a gene expression analysis of 216 snap-frozen liver biopsies, comprising 206 NAFLD cases with different fibrosis stages and 10 controls (Govaere et al, 2020; Data ref: Govaere et al, 2020) and Dataset EV1.

## Supplementary information

Appendix

Peer Review File

Dataset EV1

Source data Fig. 1

Source data Fig. 2

Source data Fig. 3

Source data Fig. 4

Source data Fig. 5

EV Figures Source Data

Expanded View Figures

## Data Availability

The RNA sequencing data from this publication have been deposited to the SRA database on NCBI and assigned the BioProject accession no. PRJNA1116241. The source data of this paper are collected in the following database record: biostudies:S-SCDT-10_1038-S44319-024-00348-7.
