# Supplementary material for: TRIM32 regulates insulin sensitivity by controlling insulin receptor degradation in the liver
Source: EMBO Rep. 2025 Jan 2;26(3):791–809. doi: 10.1038/s44319-024-00348-7 (PMC11811033; doi:10.1038/s44319-024-00348-7)
Supplement: Supplementary file 1 — Appendix [file 44319_2024_348_MOESM1_ESM.pdf]

Appendix Table of Contents

Appendix Table S1 Table showing summary of HADDOCK results of the top complexes involving TRIM32 and INSR. Page 1

Appendix Table S2 Inter-molecular contact analysis of the top two systems (system 1 and system 2 involving TRIM32 and INSR) obtained HADDOCK (A: INSR and B: TRIM32).Pages 2-3

Appendix Figure S1 Figure showing dynamics stability displaying the compactness as measured using radius of gyration and the solvent accessible surface areas of the two complexes of TRIM32-INSR over the time period of 100 ns during MD. Page 4

Appendix Figure S2 Inter-molecular contact analysis of the top ranked cluster from system 1 (A) and system 2 (B) obtained from MD simulation analysed using DIMPLOT tool of LigPlot. Page 4

Appendix Table S1. Summary of HADDOCK results of the top complexes involving TRIM32 and INSR

**HADDOCK Results**	**System 1 (Cluster 1)**	**System 2 (Cluster 1)**
HADDOCK score	-161.6 +/- 0.5	-130.6 +/- 6.1
Cluster size	186	192
RMSD from the overall lowest-energy structure	0.3 +/- 0.2	0.8 +/- 0.7
Van der Waals energy	-91.4 +/- 4.9	-61.1 +/- 4.0
Electrostatic energy	-381.4 +/- 23.4	-461.0 +/- 27.7
Desolvation Restraints violation energy	-7.3 +/- 0.7	9.1 +/- 1.1
Restraints violation energy	133.7 +/- 0.4	135.6 +/- 1.5
Buried Surface Area	2437.1 +/- 23.2	2254.9 +/- 52.8
Z-Score	-1.61	-1.92

Appendix Table S2 : Inter-molecular contact analysis of the top two systems (system 1 and system 2 involving TRIM32 and INSR) obtained HADDOCK (A: INSR and B: TRIM32).

**Interacting Pairs (System 1)**	**Distance**	**Type of bond**	**Category**
A: LYS1085:HZ2 - B: GLU11:OE2	3.26	Hydrogen Bond; Electrostatic	Salt Bridge; Attractive Charge
A:ARG1089:HH21 - B:GLU11:OE1	1.65	Hydrogen Bond; Electrostatic	Salt Bridge; Attractive Charge
A:ARG1092:HH11 - B:GLU14:OE2	1.57	Hydrogen Bond; Electrostatic	Salt Bridge; Attractive Charge
A:ARG1092:HH22 - B:GLU14:OE2	2.74	Hydrogen Bond; Electrostatic	Salt Bridge; Attractive Charge
B:ARG55:HH12 - A:ASP1232:OD2	1.90	Hydrogen Bond; Electrostatic	Salt Bridge; Attractive Charge
B:ARG55:HH21 - A:ASP1232:OD2	1.68	Hydrogen Bond; Electrostatic	Salt Bridge; Attractive Charge
B:LYS61:HZ1 - A:ASP1229:OD1	2.49	Hydrogen Bond; Electrostatic	Salt Bridge; Attractive Charge
B:LYS61:HZ3 - A:ASP1229:OD2	1.57	Hydrogen Bond; Electrostatic	Salt Bridge; Attractive Charge
A:LYS1085:NZ - B:ASP7:OD1	5.59	Electrostatic	Attractive Charge
A:ARG1089:NH1 - B:GLU11:OE2	5.22	Electrostatic	Attractive Charge
A:ARG1089:NH1 - B:GLU14:OE1	5.21	Electrostatic	Attractive Charge
A:ARG1089:NH2 - B:ASP7:OD1	5.08	Electrostatic	Attractive Charge
A:ARG1092:NH2 - B:GLU11:OE2	4.80	Electrostatic	Attractive Charge
A:ARG1092:NH2 - B:GLU14:OE1	5.06	Electrostatic	Attractive Charge
A:ARG1136:NH1 - B:ASP7:OD1	4.90	Electrostatic	Attractive Charge
B:SER2:N - A:GLU1216:OE2	4.34	Electrostatic	Attractive Charge
B:ARG55:NH1 - A:ASP1232:OD1	3.86	Electrostatic	Attractive Charge
A:ARG1089:HE - B:GLU11:OE2	2.39	Hydrogen Bond	Hydrogen Bond
A:ARG1092:HH12 - B:MET19:SD	2.09	Hydrogen Bond	Hydrogen Bond
A:GLN1208:HE21 - B:ALA8:O	2.96	Hydrogen Bond	Hydrogen Bond
A:GLN1211:HE22 - B:GLU11:O	3.01	Hydrogen Bond	Hydrogen Bond
A:ASN1215:HD21 - B:HIS3:O	1.78	Hydrogen Bond	Hydrogen Bond
A:ASP1232:HN - B:PHE58:O	1.75	Hydrogen Bond	Hydrogen Bond
B:HIS3:HN - A:GLU1216:OE2	2.31	Hydrogen Bond	Hydrogen Bond
B:HIS3:HE2 - A:GLY1169:O	2.89	Hydrogen Bond	Hydrogen Bond
B:CYS18:HG - A:GLU1094:OE2	1.58	Hydrogen Bond	Hydrogen Bond
A:LYS1168:NZ - B:HIS3	3.70	Electrostatic	Pi-Cation
A:GLU1207:OE1 - B:PHE58	3.69	Electrostatic	Pi-Anion
A:GLU1216:OE2 - B:HIS3	4.39	Electrostatic	Pi-Anion
A:ALA1095 - B:MET19	4.97	Hydrophobic	Alkyl
A:LEU1170 - B:LEU4	5.21	Hydrophobic	Alkyl
B:HIS3 - A:LYS1168	4.98	Hydrophobic	Pi-Alkyl
B:HIS3 - A:LEU1171	5.02	Hydrophobic	Pi-Alkyl
**Interacting Pairs (System 2)**	**Distance**	**Type of bond**	**Category**
A:ARG1101:HH12 - B:ASP73:OD2	2.06	Hydrogen Bond; Electrostatic	Salt Bridge; Attractive Charge
B:SER2:HN - A:GLU1001:OE1	1.68	Hydrogen Bond; Electrostatic	Salt Bridge; Attractive Charge
B:LYS61:HZ2 - A:GLU1022:OE2	1.68	Hydrogen bond; Electrostatic	Salt Bridge; Attractive Charge

B:LYS61:HZ3 - A:GLU1022:OE1	2.29	Hydrogen bond; Electrostatic	Salt Bridge; Attractive Charge
B:LYS79:HZ1 - A:GLU1096:OE1	2.53	Hydrogen bond; Electrostatic	Salt Bridge; Attractive Charge
B:LYS79:HZ2 - A:GLU1096:OE2	1.65	Hydrogen bond; Electrostatic	Salt Bridge; Attractive Charge
A:ARG1000:HH22 - B:ASN74:OD1	2.97	Hydrogen Bond	Hydrogen Bond
A:ASN1014:HD22 - B:HIS36:NE2	2.05	Hydrogen Bond	Hydrogen Bond
A:ALA1023:HN - B:PHE58:O	2.14	Hydrogen Bond	Hydrogen Bond
A:GLU1024:HN - B:PHE58:O	2.24	Hydrogen Bond	Hydrogen Bond
A:ARG1026:HN - B:CYS34:O	2.87	Hydrogen Bond	Hydrogen Bond
A:ARG1026:HH12 - B:GLU11:O	1.93	Hydrogen Bond	Hydrogen Bond
A:ARG1026:HH22 - B:GLU11:O	2.36	Hydrogen Bond	Hydrogen Bond
B:HIS3:HN - A:GLU1001:OE1	1.78	Hydrogen Bond	Hydrogen Bond
B:LEU4:HN - A:GLU1001:OE1	2.40	Hydrogen Bond	Hydrogen Bond
B:ASN5:HN - A:LEU999:O	2.46	Hydrogen Bond	Hydrogen Bond
B:ASN5:HD21 - A:LEU998:O	2.22	Hydrogen Bond	Hydrogen Bond
B:ARG28:HH22 - A:PRO1099:O	2.89	Hydrogen Bond	Hydrogen Bond
B:CYS59:HG - A:GLU1024:O	1.79	Hydrogen Bond	Hydrogen Bond
B:SER60:HN - A:GLU1022:OE2	2.89	Hydrogen Bond	Hydrogen Bond
B:SER60:HG - A:GLY1021:O	1.71	Hydrogen Bond	Hydrogen Bond
B:LYS61:HN - A:GLU1022:OE2	2.11	Hydrogen Bond	Hydrogen Bond
A:ASP1143:OD2 - B:HIS33	4.87	Electrostatic	Pi-Anion
B:ASP73:HN - A:HIS1081	3.07	Hydrogen Bond	Pi-Donor Hydrogen Bond
B:HIS33:C,O;CYS34:N - A:HIS1142	3.91	Hydrophobic	Amide-Pi Stacked
A:ARG1000 - B:VAL12	3.84	Hydrophobic	Alkyl
A:ALA1080 - B:LEU31	3.57	Hydrophobic	Alkyl
B:ALA8 - A:LEU999	4.27	Hydrophobic	Alkyl
A:HIS1081 - B:LEU75	4.92	Hydrophobic	Pi-Alkyl

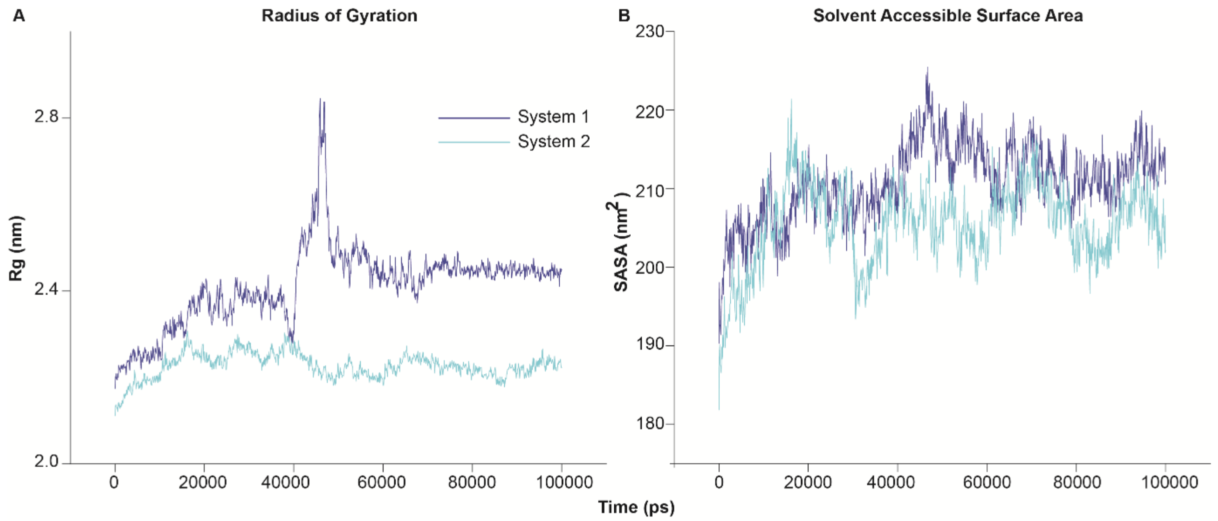

Appendix Figure S1. Dynamics stability displaying the compactness as measured using radius of gyration and the solvent accessible surface areas of the two complexes of TRIM32-INSR over the time period of 100 ns during MD.

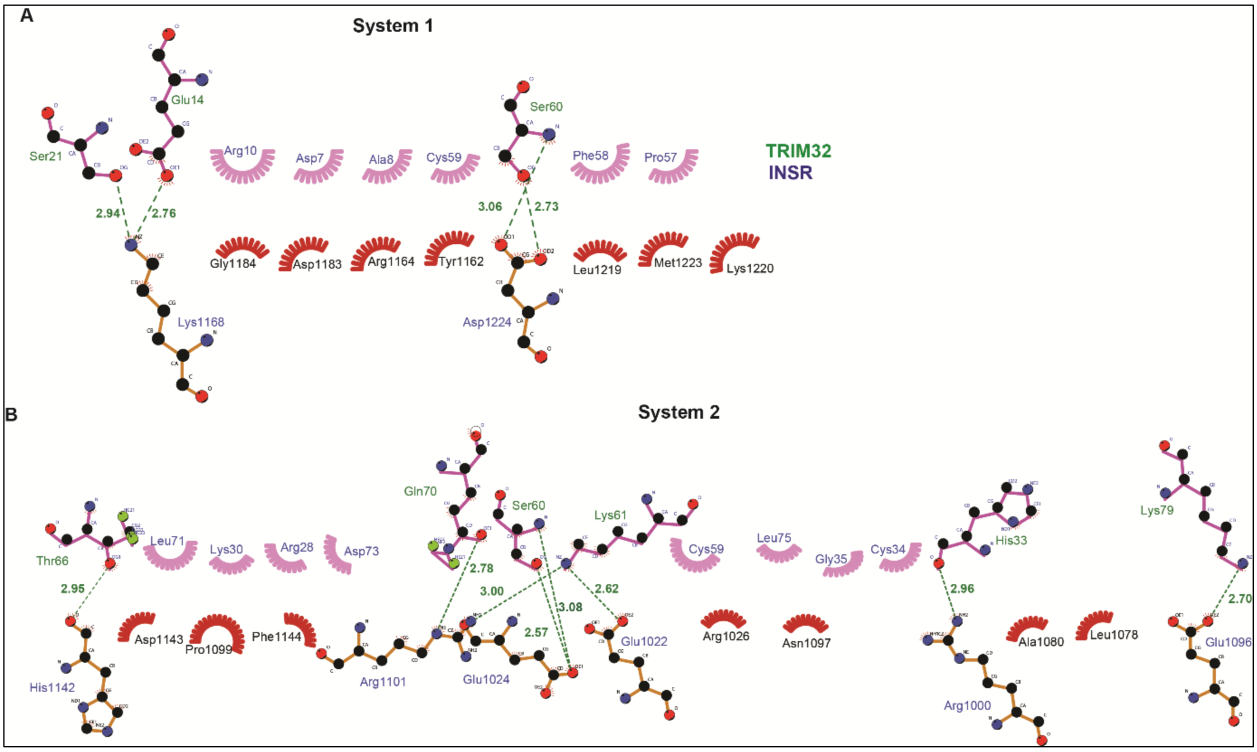

Appendix Figure S2. Inter-molecular contact analysis of the top ranked cluster from system 1 (A) and system 2 (B) obtained from MD simulation analysed using DIMPLOT tool of LigPlot$^+$. The dashed lines indicate hydrogen bonds and their distances between interacting pairs of amino acids.
