# Supplementary figures and images for: TRIM32 regulates insulin sensitivity by controlling insulin receptor degradation in the liver
Source: EMBO Rep. 2025 Jan 2;26(3):791–809. doi: 10.1038/s44319-024-00348-7 (PMC11811033; doi:10.1038/s44319-024-00348-7)

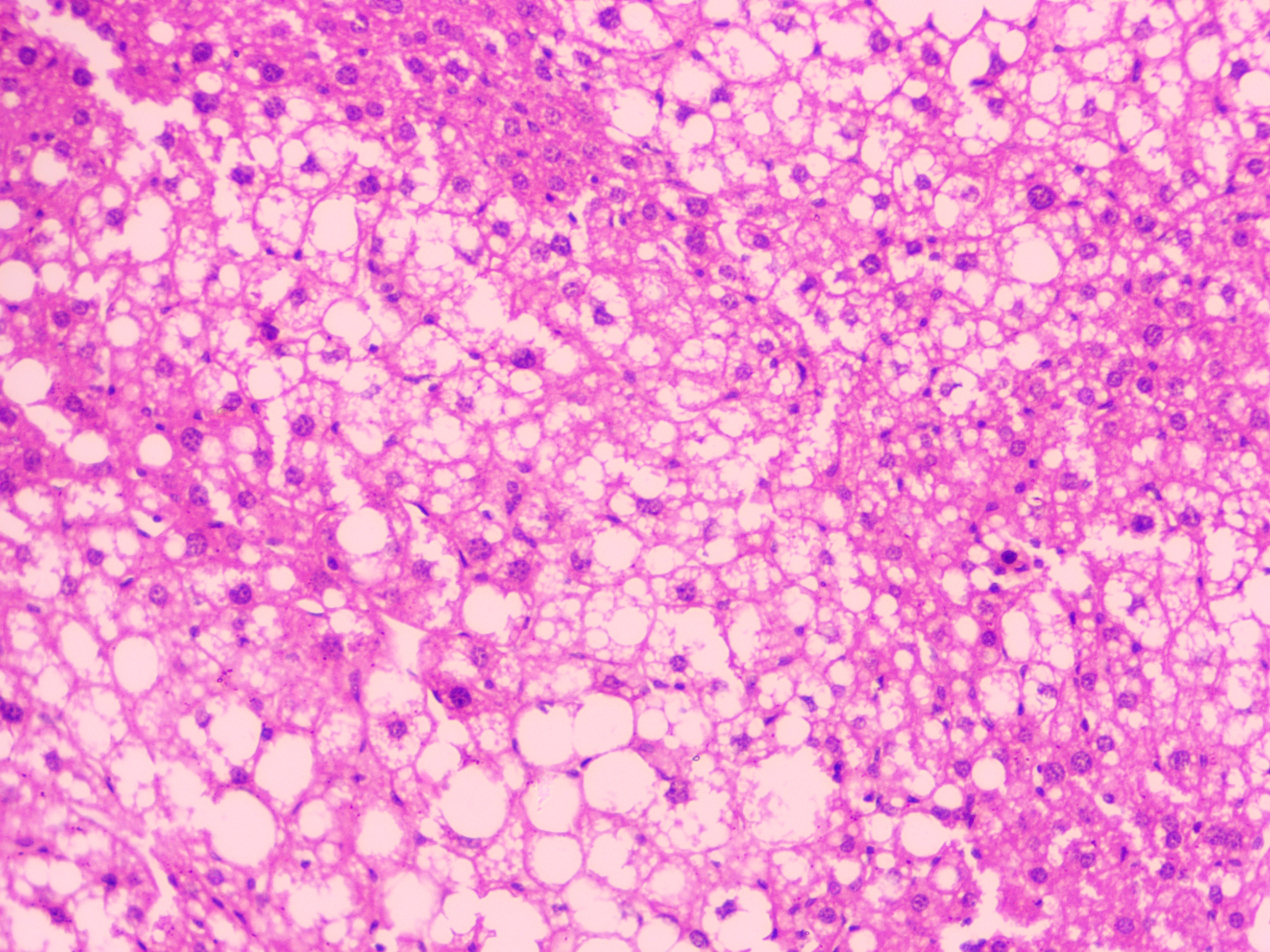

Supplement: Supplementary file 4 — Source data Fig. 1 [file 44319_2024_348_MOESM4_ESM.zip › Figure1/1G/H&E/HFD liver.tiff]

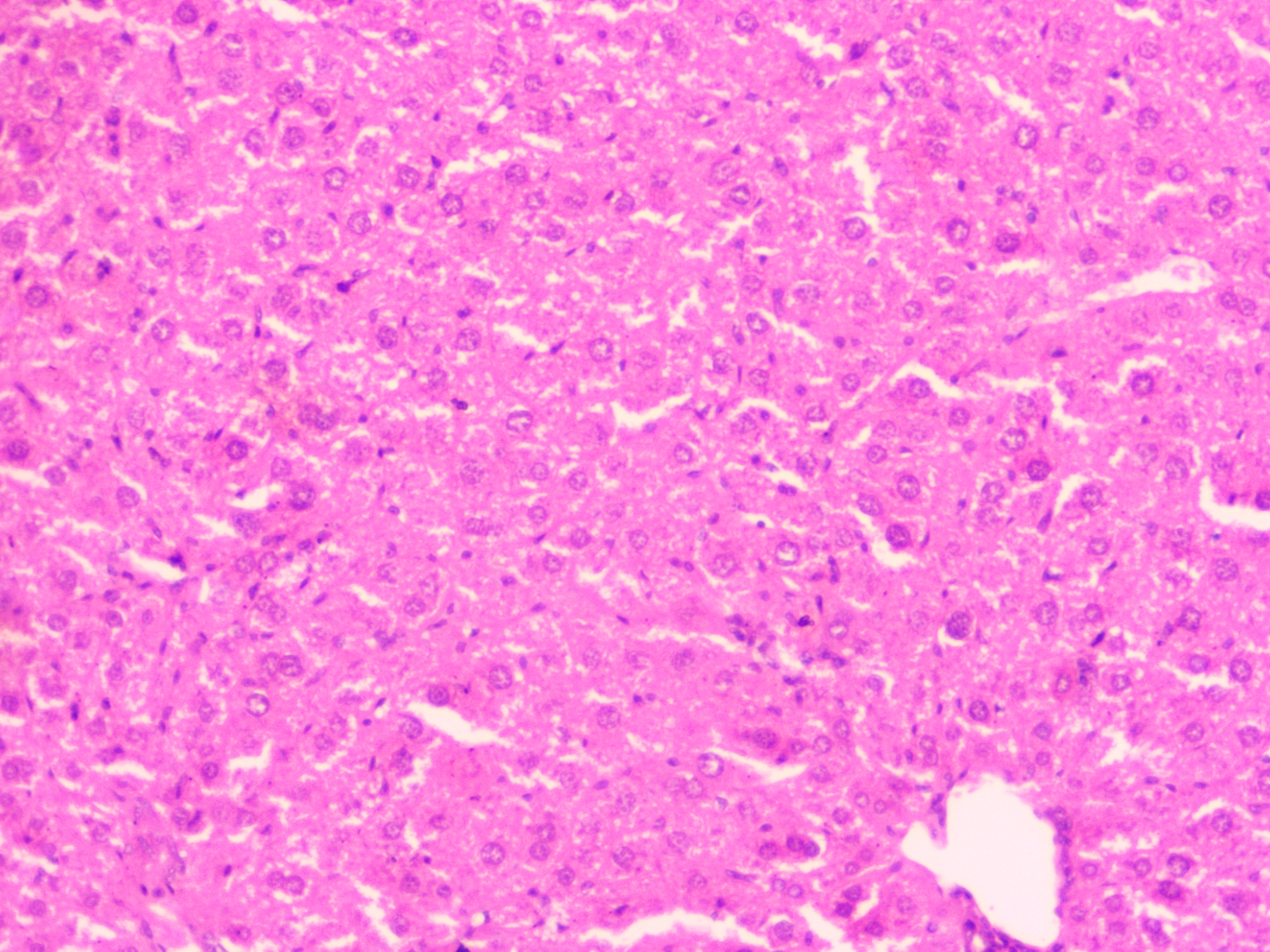

Supplement: Supplementary file 4 — Source data Fig. 1 [file 44319_2024_348_MOESM4_ESM.zip › Figure1/1G/H&E/RCD liver.tiff]

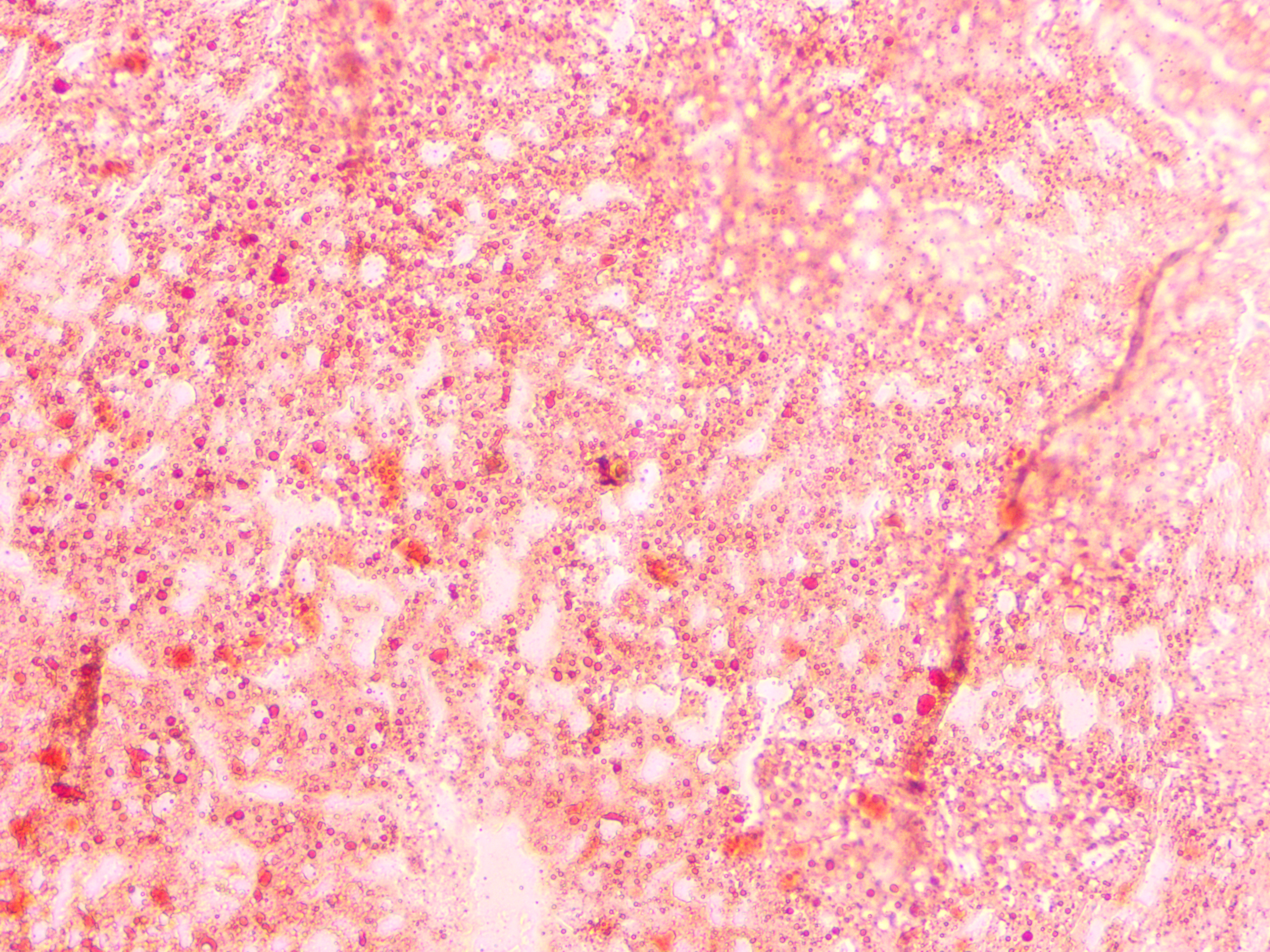

Supplement: Supplementary file 4 — Source data Fig. 1 [file 44319_2024_348_MOESM4_ESM.zip › Figure1/1G/Oil red O/HFD liver.tiff]

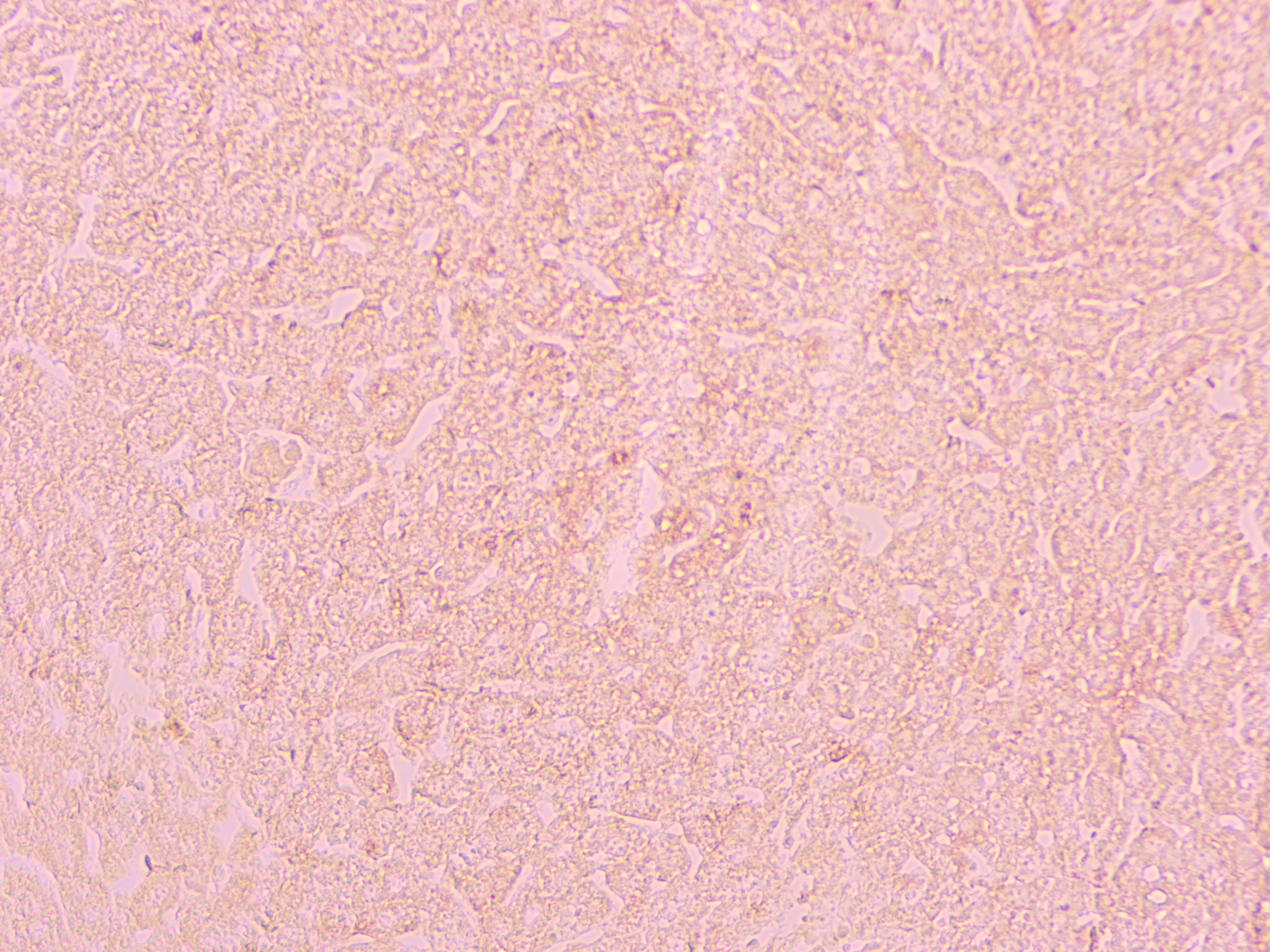

Supplement: Supplementary file 4 — Source data Fig. 1 [file 44319_2024_348_MOESM4_ESM.zip › Figure1/1G/Oil red O/RCD liver.tiff]

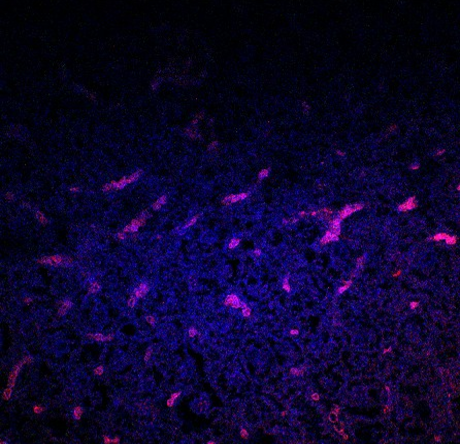

Supplement: Supplementary file 4 — Source data Fig. 1 [file 44319_2024_348_MOESM4_ESM.zip › Figure1/1G/SIRIUS RED/hfd1 liver merged.tif]

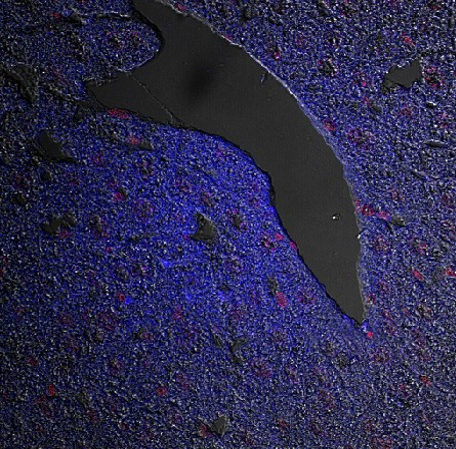

Supplement: Supplementary file 4 — Source data Fig. 1 [file 44319_2024_348_MOESM4_ESM.zip › Figure1/1G/SIRIUS RED/rcd1 liver merged.tif]

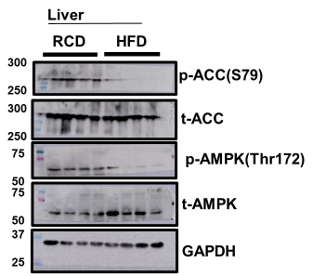

Supplement: Supplementary file 4 — Source data Fig. 1 [file 44319_2024_348_MOESM4_ESM.zip › Figure1/1H/1H_western blot.tif]

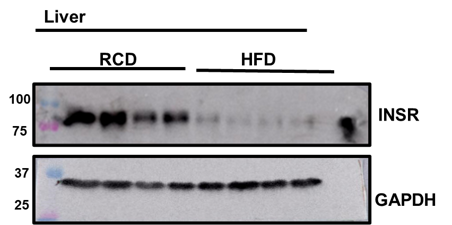

Supplement: Supplementary file 4 — Source data Fig. 1 [file 44319_2024_348_MOESM4_ESM.zip › Figure1/1I/1I_western blot.tif]

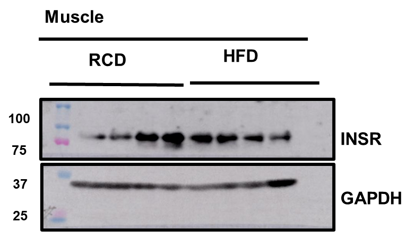

Supplement: Supplementary file 4 — Source data Fig. 1 [file 44319_2024_348_MOESM4_ESM.zip › Figure1/1J/1J_western blot.tif]

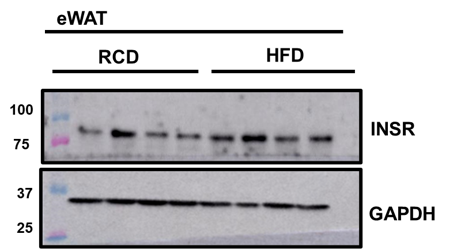

Supplement: Supplementary file 4 — Source data Fig. 1 [file 44319_2024_348_MOESM4_ESM.zip › Figure1/1K/1K_western blot.tif]

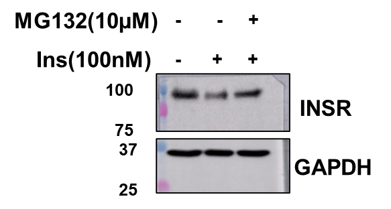

Supplement: Supplementary file 5 — Source data Fig. 2 [file 44319_2024_348_MOESM5_ESM.zip › Figure2/2A/2A_western blot.tif]

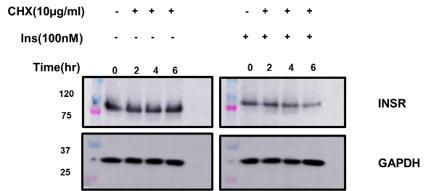

Supplement: Supplementary file 5 — Source data Fig. 2 [file 44319_2024_348_MOESM5_ESM.zip › Figure2/2B/2B_western blot.tif]

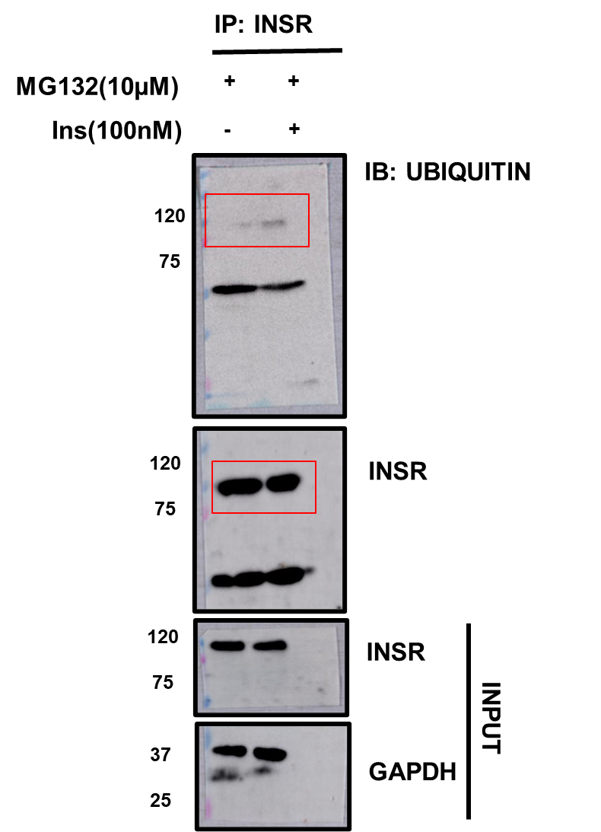

Supplement: Supplementary file 5 — Source data Fig. 2 [file 44319_2024_348_MOESM5_ESM.zip › Figure2/2C/2C_western blot.tif]

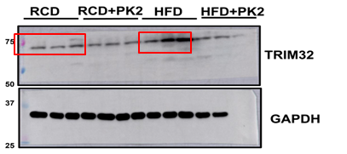

Supplement: Supplementary file 5 — Source data Fig. 2 [file 44319_2024_348_MOESM5_ESM.zip › Figure2/2G/2G_western blot.2.tif]

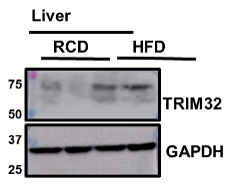

Supplement: Supplementary file 5 — Source data Fig. 2 [file 44319_2024_348_MOESM5_ESM.zip › Figure2/2G/2G_western blot.tif]

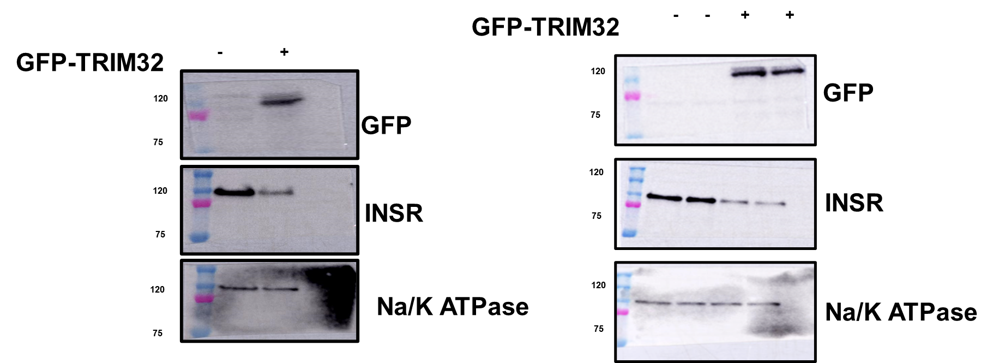

Supplement: Supplementary file 5 — Source data Fig. 2 [file 44319_2024_348_MOESM5_ESM.zip › Figure2/2H/2H_western blot.tif]

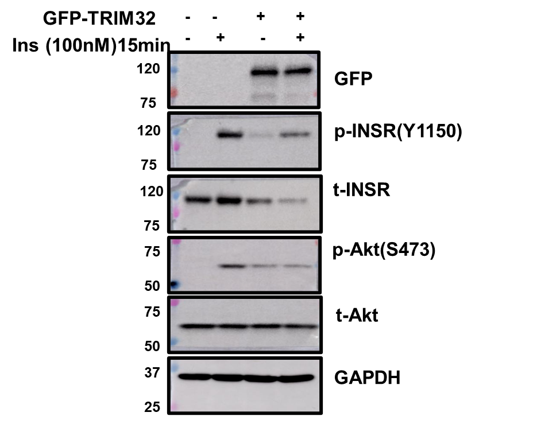

Supplement: Supplementary file 6 — Source data Fig. 3 [file 44319_2024_348_MOESM6_ESM.zip › Figure3/3A/3A_western blot.tif]

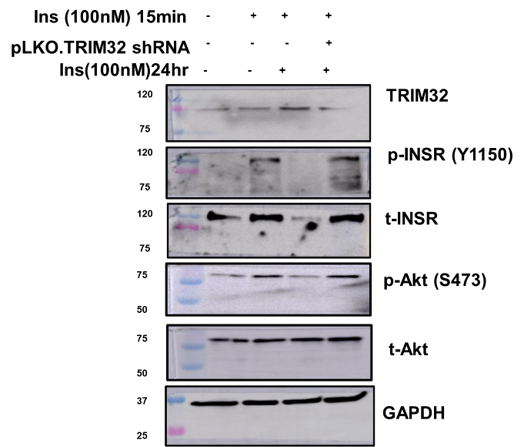

Supplement: Supplementary file 6 — Source data Fig. 3 [file 44319_2024_348_MOESM6_ESM.zip › Figure3/3B/3B_western blot.tif]

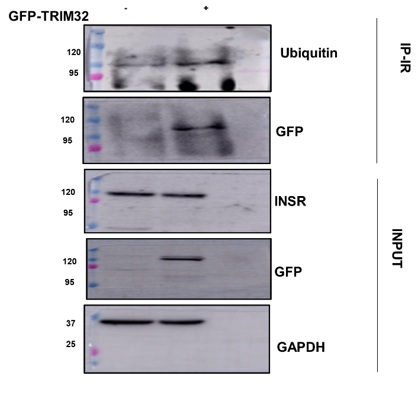

Supplement: Supplementary file 6 — Source data Fig. 3 [file 44319_2024_348_MOESM6_ESM.zip › Figure3/3C/3C_western blot.tif]

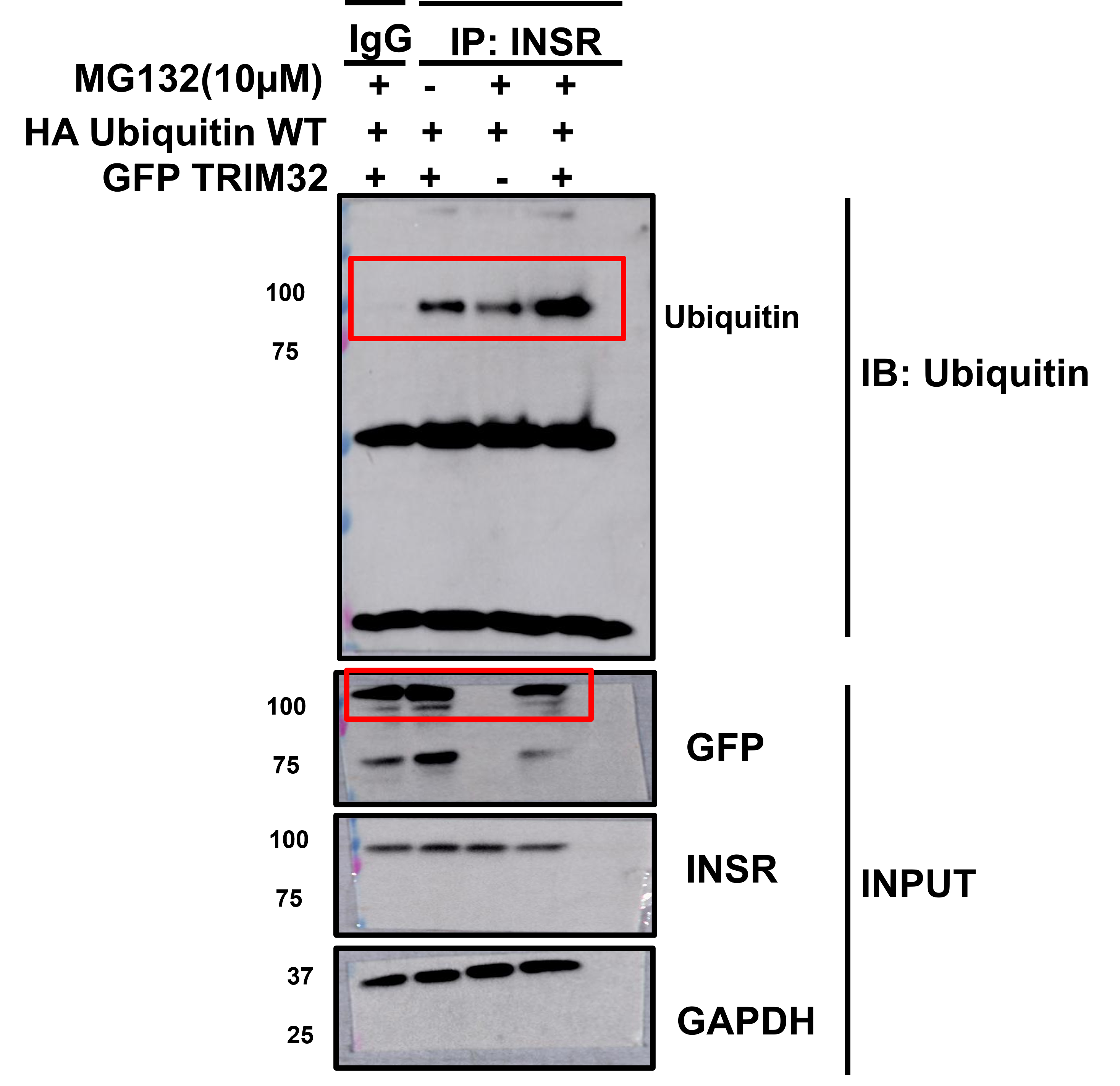

Supplement: Supplementary file 6 — Source data Fig. 3 [file 44319_2024_348_MOESM6_ESM.zip › Figure3/3D/3D_western blot.tif]

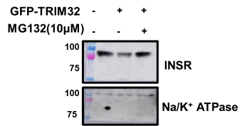

Supplement: Supplementary file 6 — Source data Fig. 3 [file 44319_2024_348_MOESM6_ESM.zip › Figure3/3E/3E_western blot.tif]

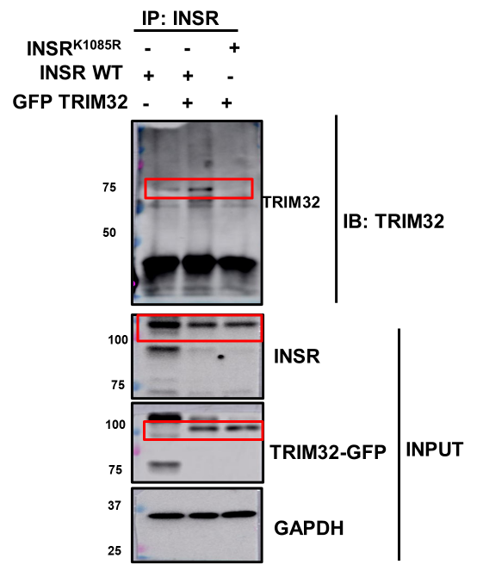

Supplement: Supplementary file 6 — Source data Fig. 3 [file 44319_2024_348_MOESM6_ESM.zip › Figure3/3I/3I_western blot.tif]

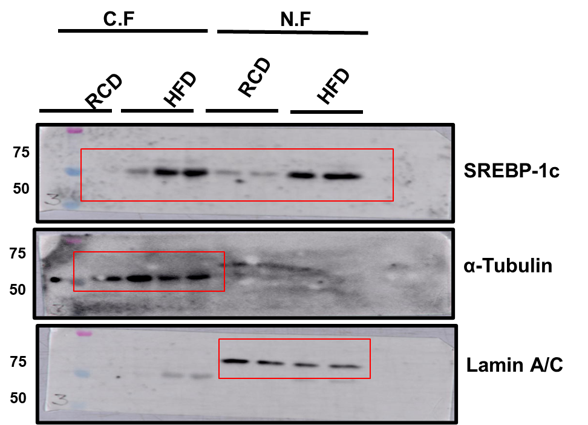

Supplement: Supplementary file 7 — Source data Fig. 4 [file 44319_2024_348_MOESM7_ESM.zip › Figure4/4C/4C_western blot.tif]

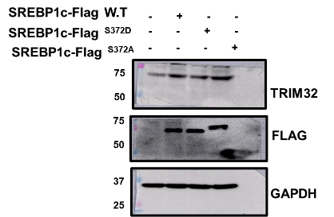

Supplement: Supplementary file 7 — Source data Fig. 4 [file 44319_2024_348_MOESM7_ESM.zip › Figure4/4D/4D_western blot.tif]

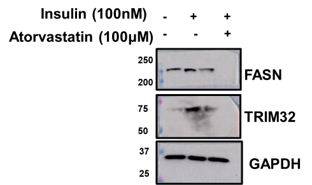

Supplement: Supplementary file 7 — Source data Fig. 4 [file 44319_2024_348_MOESM7_ESM.zip › Figure4/4E/4E_western blot.tif]

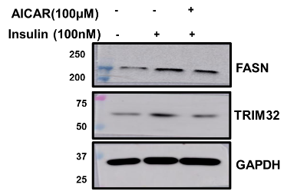

Supplement: Supplementary file 7 — Source data Fig. 4 [file 44319_2024_348_MOESM7_ESM.zip › Figure4/4F/4F_western blot.tif]

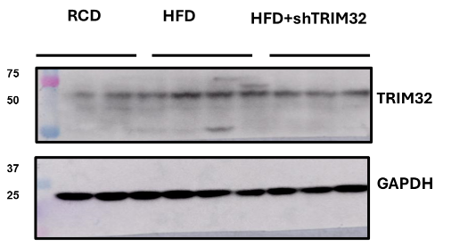

Supplement: Supplementary file 8 — Source data Fig. 5 [file 44319_2024_348_MOESM8_ESM.zip › Figure5/5B/5B_western blot.tif]

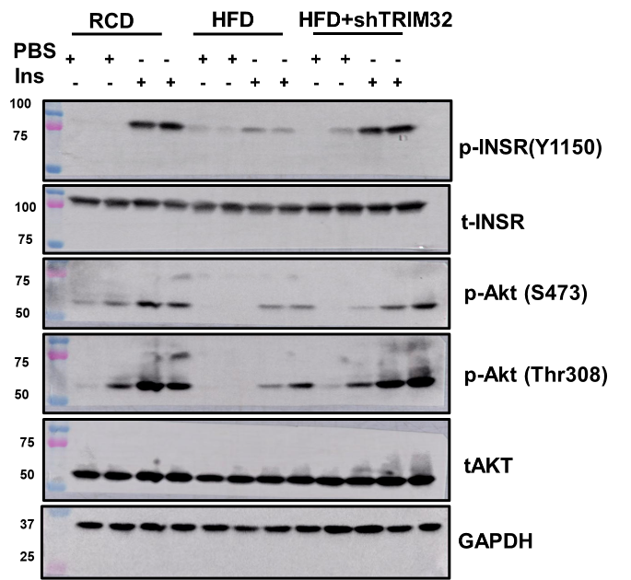

Supplement: Supplementary file 8 — Source data Fig. 5 [file 44319_2024_348_MOESM8_ESM.zip › Figure5/5E/5E_western blot.tif]

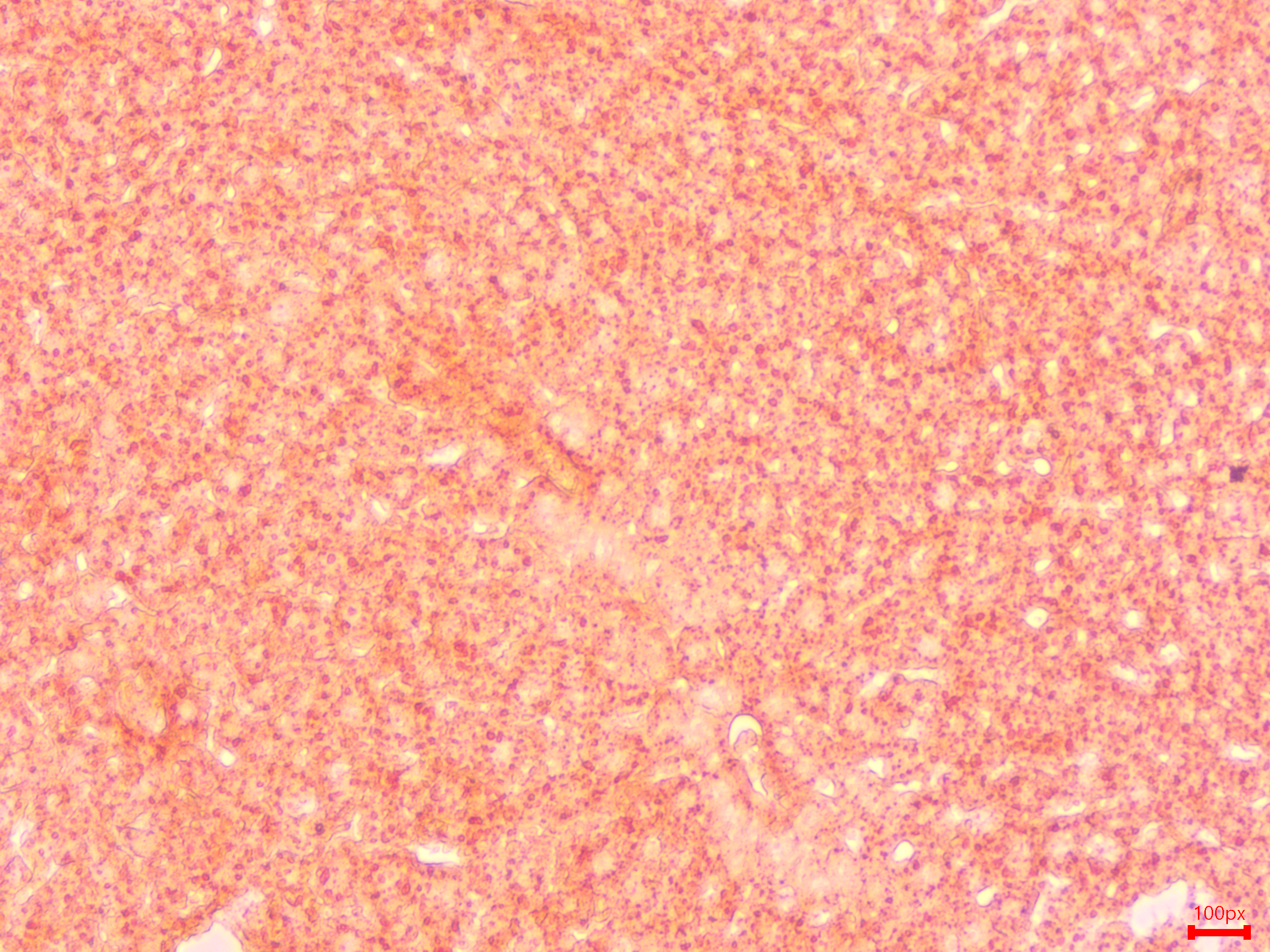

Supplement: Supplementary file 8 — Source data Fig. 5 [file 44319_2024_348_MOESM8_ESM.zip › Figure5/5M/HFD+shTRIM32.2.tif]

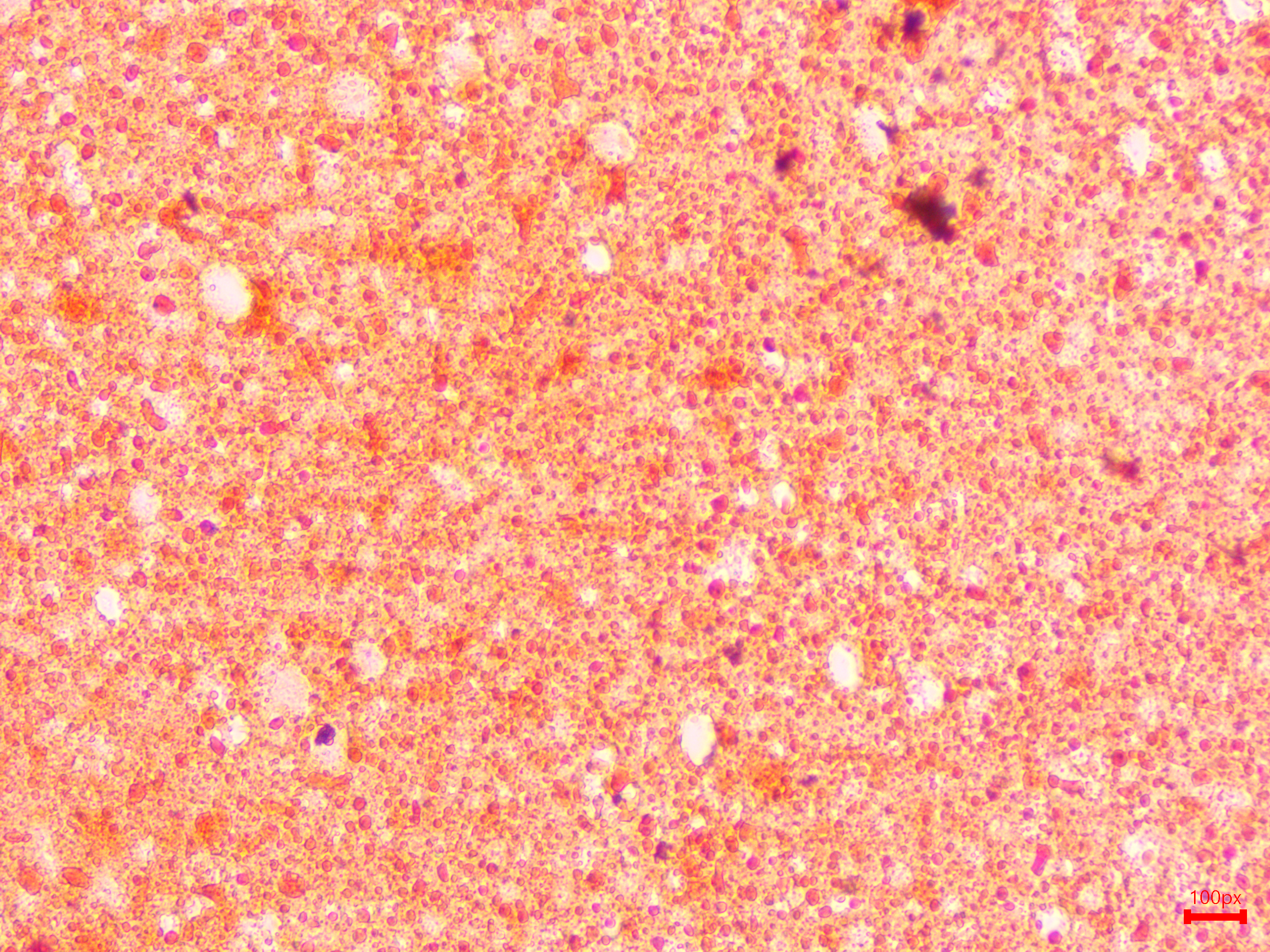

Supplement: Supplementary file 8 — Source data Fig. 5 [file 44319_2024_348_MOESM8_ESM.zip › Figure5/5M/HFD1.tiff]

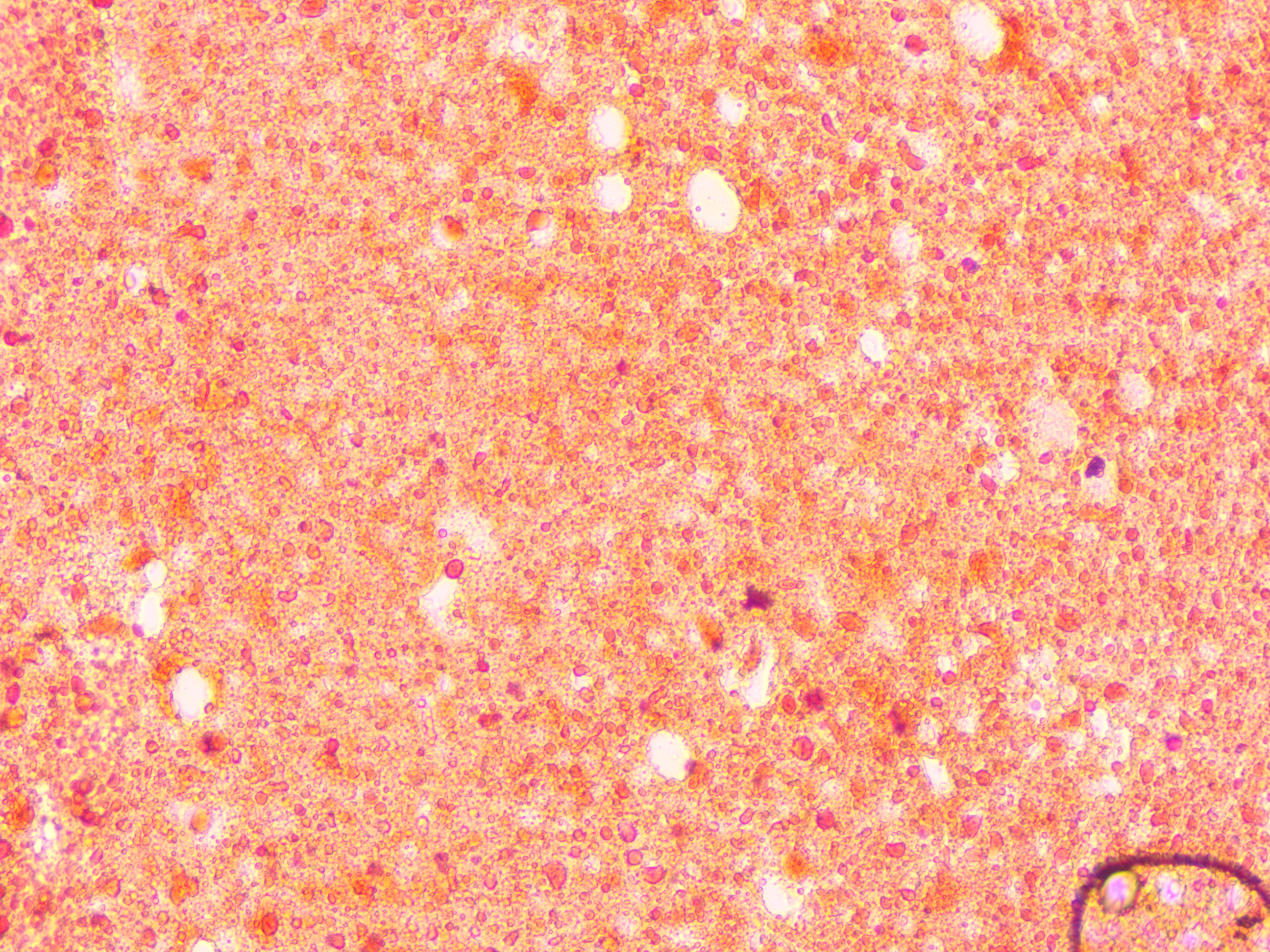

Supplement: Supplementary file 8 — Source data Fig. 5 [file 44319_2024_348_MOESM8_ESM.zip › Figure5/5M/HFD2.tiff]

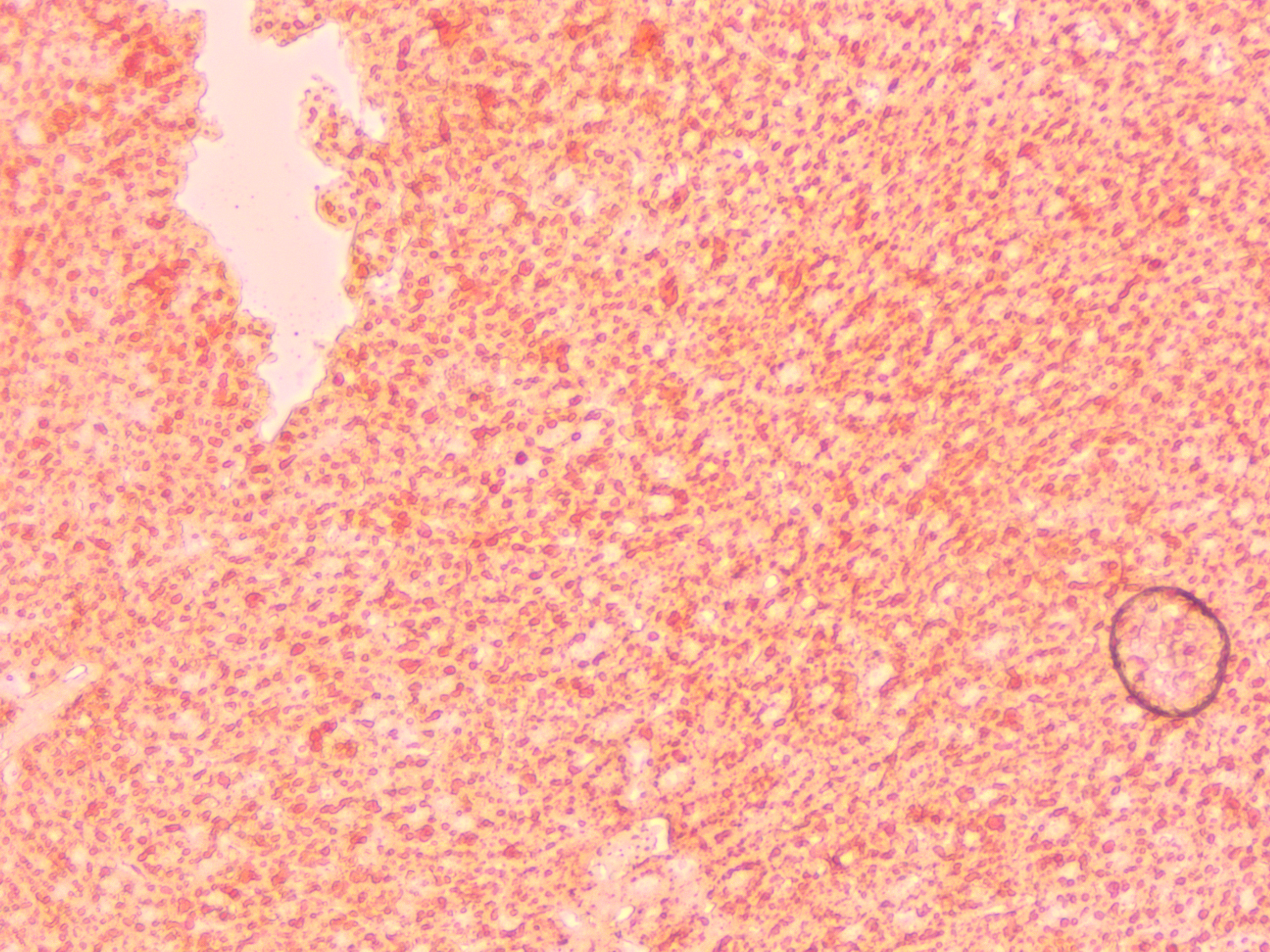

Supplement: Supplementary file 8 — Source data Fig. 5 [file 44319_2024_348_MOESM8_ESM.zip › Figure5/5M/HFD_shTRIM32.1.tiff]

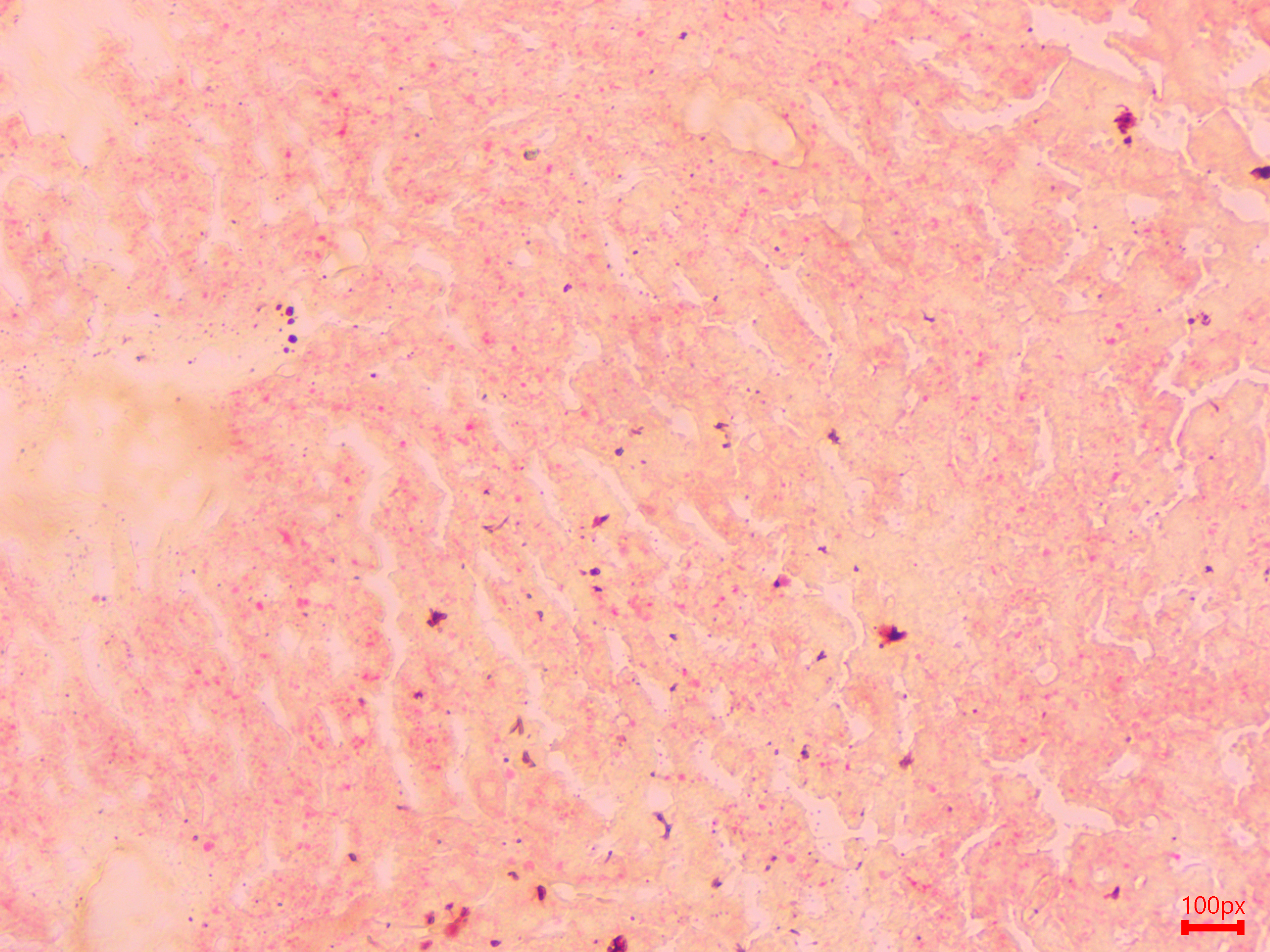

Supplement: Supplementary file 8 — Source data Fig. 5 [file 44319_2024_348_MOESM8_ESM.zip › Figure5/5M/RCD.tiff]

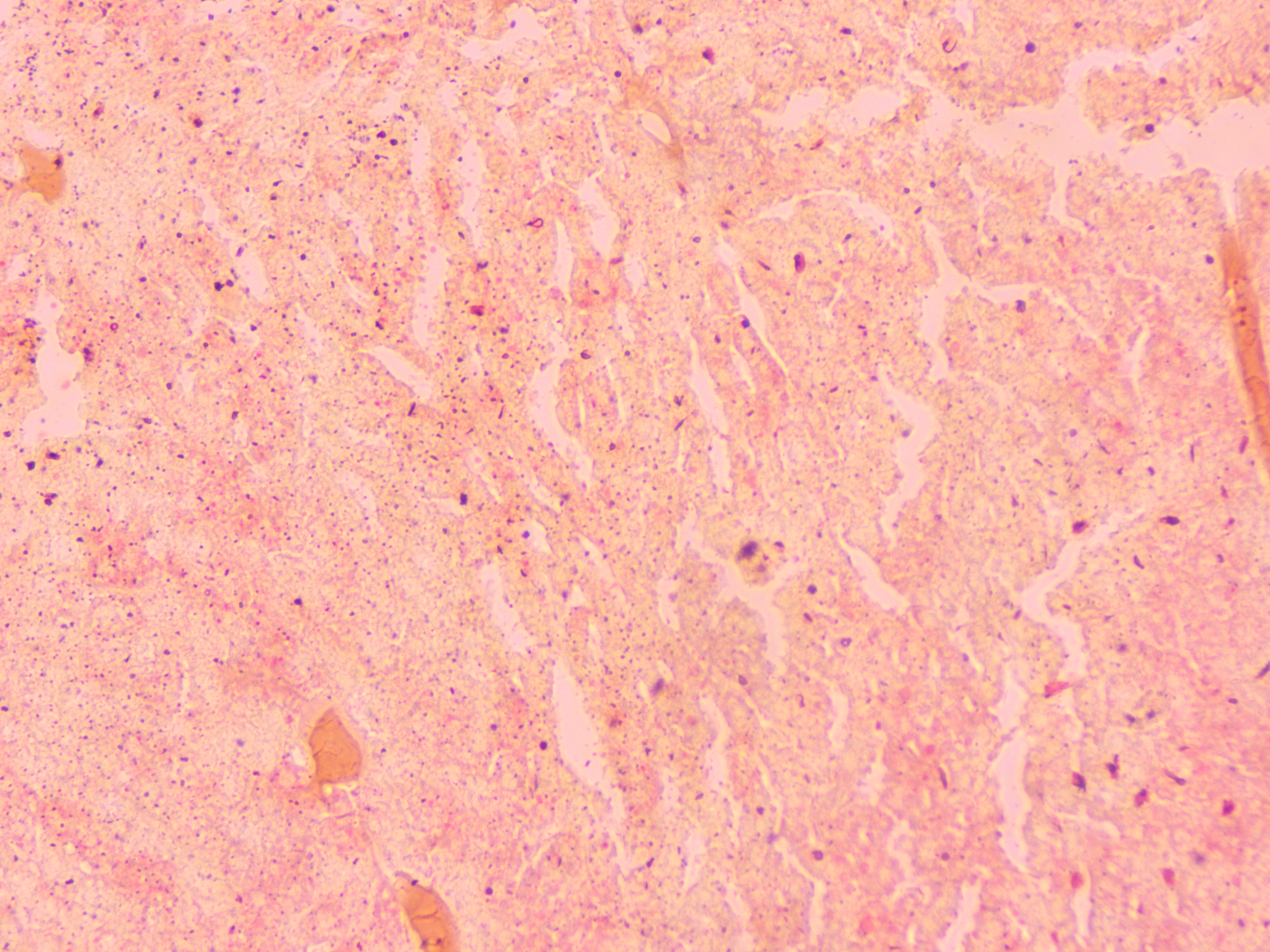

Supplement: Supplementary file 8 — Source data Fig. 5 [file 44319_2024_348_MOESM8_ESM.zip › Figure5/5M/RCD1.tiff]

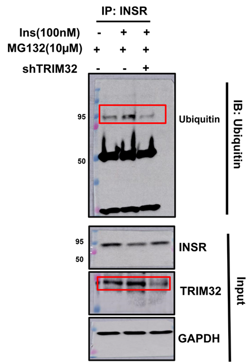

Supplement: Supplementary file 9 — EV Figures Source Data [file 44319_2024_348_MOESM9_ESM.zip › EV figures/EV figure3/3E/EV3E_western blot.tif]
