# Supplementary material for: TRIM32 regulates insulin sensitivity by controlling insulin receptor degradation in the liver
Source: EMBO Rep. 2025 Jan 2;26(3):791–809. doi: 10.1038/s44319-024-00348-7 (PMC11811033; doi:10.1038/s44319-024-00348-7)
Supplement: Supplementary file 10 — Expanded View Figures [file 44319_2024_348_MOESM10_ESM.pdf]

Expanded View Figures

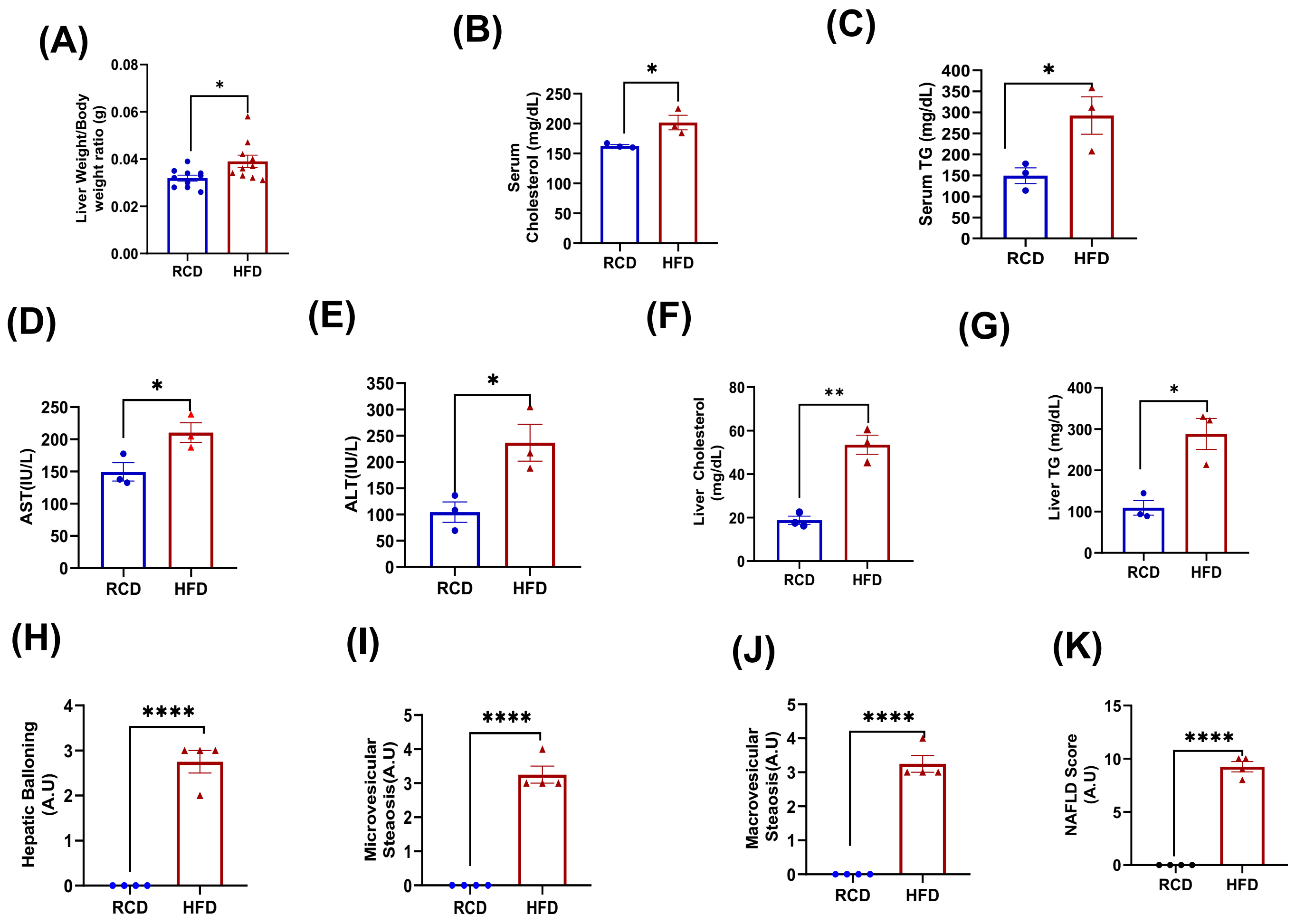

Figure EV1. High-fat diet-induced insulin resistance is primarily mediated by the degradation of hepatic insulin receptors.

(**A**) Graphical representation of normalized Liver to body weight ratio ($n = 10$). (**B–E**) Graphical representation of serum cholesterol, triglyceride, AST, and ALT levels, respectively ($n = 3$), biological replicates. (**F**, **G**) Graphical representation of Liver Triglyceride and Cholesterol data, respectively ($n = 3$), biological replicates. (**H–K**) Graphical representation of hepatic ballooning, macrovesicular & microvesicular steatosis, and NAFLD score ($n = 3$), biological replicates. Graphs were plotted in Graph pad prism8. The statistical significance was assessed by unpaired two-tailed t-test, and data has been expressed as Mean ± SEM. *$p < 0.05$, **$p < 0.01$, ***$p < 0.001$, and ****$p < 0.0001$. Data information: In (1**A**) unpaired two-tailed t-test, $p = 0.0252$ (RCD vs. HFD), (**B**) RCD vs. HFD ($p = 0.034$), (**C**) RCD vs. HFD ($p = 0.0414$), (**D**) AST ratio of RCD vs. HFD ($p = 0.0420$), (**E**) ALT ratio of RCD vs. HFD ($p = 0.0009$), (**F**) Liver Cholesterol RCD vs. HFD ($p = 0.0019$), (**G**) Liver TG of RCD vs. HFD ($p = 0.0125$), (**H**) RCD vs. HFD ($p < 0.0001$), (**I**) RCD vs HFD ($p < 0.0001$), (**J**) RCD vs HFD ($p < 0.0001$), (**K**) RCD vs HFD ($p < 0.0001$). Source data are available online for this figure.

© The Author(s)

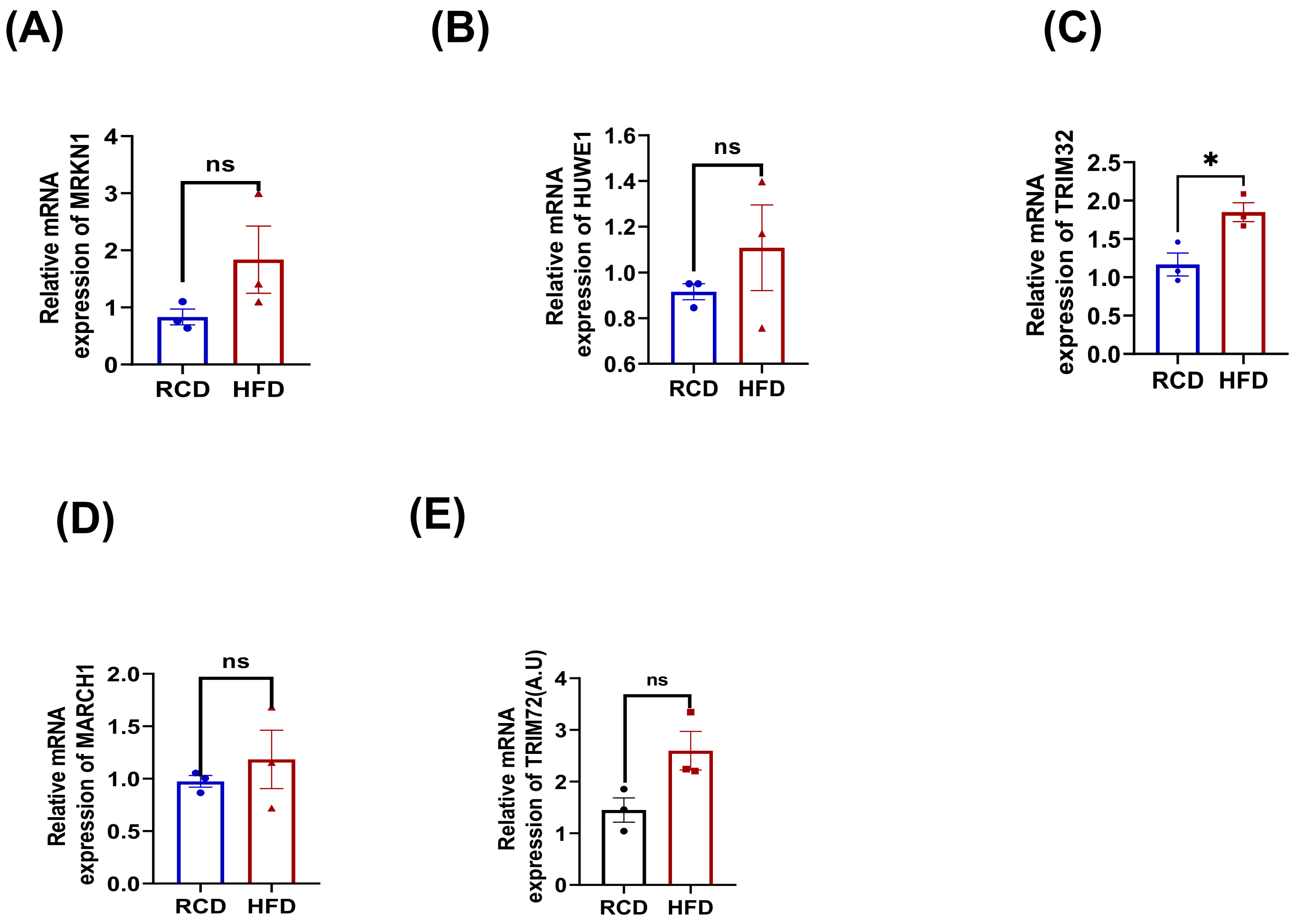

Figure EV2. HFD induces TRIM32 expression to promote hepatic insulin receptor ubiquitination and degradation.

(**A–E**) Quantitative representation of RT-PCR data for MRKN1, HUWE1, TRIM32, MARCH 1, and TRIM72 E3 ubiquitin ligases from RCD and HFD liver ($n = 3$), biological replicates. Graphs were plotted in Graph pad prism8. The statistical significance was assessed by unpaired two-tailed t-test, and the data has been expressed as Mean ± SEM. *$p < 0.05$, **$p < 0.01$, ***$p < 0.001$, and ****$p < 0.0001$. Data information: (**A**) RCD vs HFD ($p = 0.1718$), (**B**) RCD vs HFD ($p = 0.3962$), (**C**) RCD vs HFD ($p = 0.0250$), (**D**) RCD vs HFD ($p = 0.4998$), (**E**) RCD vs HFD ($p = 0.0601$). Source data are available online for this figure.

© The Author(s)

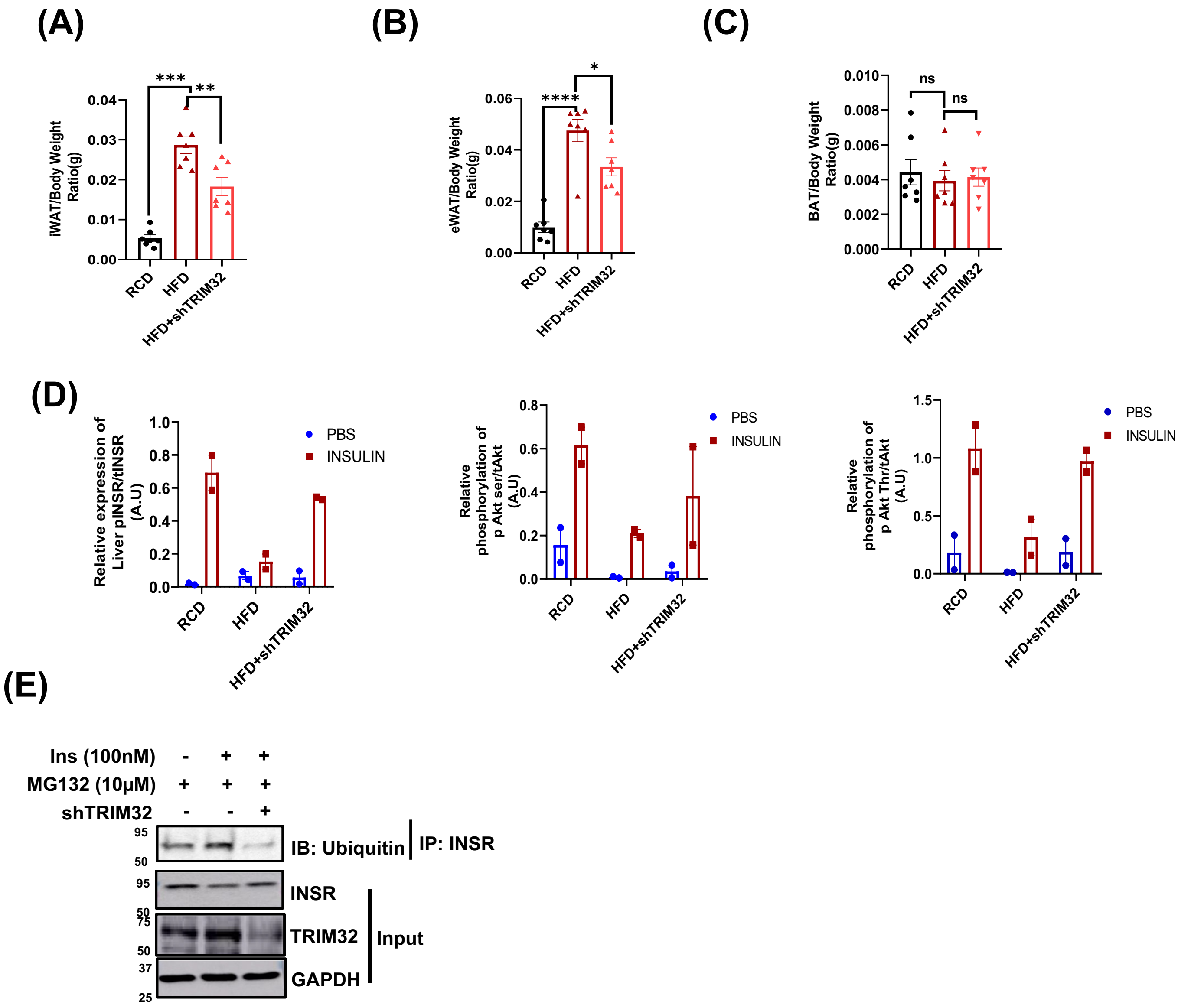

Figure EV3. Depletion of TRIM32 in the liver of HFD mice increases insulin sensitivity and reverses fatty liver.

(**A–C**) The bar graph shows the relative change of iWAT, eWAT, and BAT to body weight ratio ($n = 7$). (**D**) Quantitative representation of Western blots showing phosphorylation of INSR(Y1150) (left), Akt(S473) (middle), and Akt(Thr308) (right) ($n = 3$), biological replicates. (**E**) Mouse primary hepatocytes were transfected with the shTRIM32 plasmid and, after 24 h, co-treated with Insulin (100 nM) and MG132 (10 μM) for the next 24 h. Immunoprecipitation was performed using the INSR antibody and checked for INSR ubiquitination using WB. Blots were quantified using ImageJ software, and values were plotted in GraphPad Prism 8. The statistical significance was assessed by one-way analysis of variance (ANOVA) with Bonferroni. Data were expressed as Mean ± SEM. *$p < 0.05$, **$p < 0.01$, ***$p < 0.001$ and ****$p < 0.0001$ Data information in (**A**) iWAT by B.W. ratio of RCD vs. HFD ($p < 0.0001$) and HFD vs. HFDshTRIM32 ($p = 0.0025$), (**B**) eWAT by B.W. ratio of RCD vs. HFD ($p < 0.0001$) and HFD vs. HFDshTRIM32 ($p = 0.0287$), (**C**) BAT by B.W. ratio of RCD vs HFD ($p = 0.099$) and HFD vs HFDshTRIM32 ($p = 0.0999$). Source data are available online for this figure.
