# Supplementary material for: TRIM32 regulates insulin sensitivity by controlling insulin receptor degradation in the liver
Source: EMBO Rep. 2025 Jan 2;26(3):791–809. doi: 10.1038/s44319-024-00348-7 (PMC11811033; doi:10.1038/s44319-024-00348-7)
Supplement: Supplementary file 2 — Peer Review File [file 44319_2024_348_MOESM2_ESM.pdf]

EMBO reports

TRIM32-mediated degradation of insulin receptor

Shilpa Thakur, Priya Rawat, Budheswar Dehury, and PROSENJIT MONDAL

Corresponding author(s): PROSENJIT MONDAL (PROSENJIT@IITMANDI.AC.IN)

Review Timeline:

Submission Date:	17th Jan 24
Editorial Decision:	27th Feb 24
Revision Received:	7th Jun 24
Editorial Decision:	8th Aug 24
Revision Received:	12th Sep 24
Editorial Decision:	15th Oct 24
Revision Received:	20th Oct 24
Accepted:	13th Nov 24

Editor: Martina Rembold / Deniz Senyilmaz Tiebe

Transaction Report: No Peer Review Process File is available with this article, as the authors have chosen not to make the review process public.
